# Auditory brainstem responses in aging dark agouti rats

**DOI:** 10.1042/BSR20202724

**Published:** 2021-02-19

**Authors:** Angela K. Beltrame, Nancy M. Dahms, Christina L. Runge

**Affiliations:** 1 Department of Biochemistry, Medical College of Wisconsin, Milwaukee, Wisconsin, U.S.A.; 2 Department of Otolaryngology and Communication Sciences, Medical College of Wisconsin, Milwaukee, Wisconsin, U.S.A.

**Keywords:** auditory brainstem response, cochlea, Dark agouti, hearing, rat model

## Abstract

The present study examined auditory function across age in the dark agouti (DA) rat strain. Auditory brainstem responses (ABRs) were measured for frequencies 8, 16, and 32 kHz in male and female DA rats from 3 to 18 months of age. Hearing thresholds and absolute and interpeak latencies (IPLs) were analyzed. Male hearing thresholds remained stable for the first year of life and then significantly increased at 18 months across all frequencies; female hearing remained stable at all tested ages out to 18 months. At 12 months, male DA rats showed significantly longer absolute latencies by age (i.e., compared with 3-month-old males) and sex (compared with 12-month-old females), with no differences in IPLs. At 18 months, female DA rats showed significantly longer absolute latencies with age (compared with 3-month-old females) and sex (compared with 18-month-old males), particularly for the later waves. Female IPLs were also significantly longer with age and by sex for the later waves. This report supports the feasibility of using male DA rats in studies to investigate age-related hearing loss (ARHL; presbycusis).

## Introduction

Hearing loss is the third most common chronic disability and surveys by the Centers for Disease Control and Prevention (CDC, Atlanta, GA) reveal that it currently affects 16% of U.S. adults aged 18 and over [1]. There is a tremendous financial burden associated with hearing loss; in 2017, the World Health Organization predicted that the annual cost of unaddressed hearing loss will reach $790 billion globally [2]. One of the major causes of hearing loss is from the normal aging process, i.e., presbycusis, characterized by reduced hearing sensitivity from age-related deterioration of inner ear sensory cell, vascular and neural function [3]. As the population ages, the number of people affected with hearing loss is expected to continuously rise; data from the National Health and Nutrition Examination Survey predict an increase from 44.1 million Americans in 2020 to 73.5 million Americans in 2060 [4]. Gender differences in hearing loss, especially with presbycusis, have long been identified and described. Hearing impairment has been identified at earlier ages in men than women, decline in hearing sensitivity occurs twice as fast for men, and hearing thresholds in elderly men were identified to be higher than elderly women [5,6].

To understand the mechanisms of hearing loss and develop therapies and treatments, animal models, including rats, can be a useful tool. Several rat strains, such as Wistar, Long–Evans (LE), Sprague–Dawley (SD), and Fischer 344 (F344) rats, have been extensively studied and used for assessment of normal and pathological conditions, including hearing loss [7,8]. There are few published studies that have explored sex differences in rat hearing thresholds. Typically, rat studies have only explored the hearing of male rats [9–12] or did not identify sex differences [13,14]. Of those that have examined sex differences, one study of 1–2-month-old LE rats by Charlton et al. identified that male rats have significantly higher hearing thresholds than females at both low (1 and 4 kHz) and high (32 and 42 kHz) frequencies [15]. A study of F344 rats by Balogova et al. determined that male rats had higher hearing thresholds for frequencies ranging from 2 to 40 kHz, and developed hearing loss earlier (after 3 months) than female rats (after 8 months) [16]. Additionally, hearing loss progressed more slowly for females than males until the females reached 27–30 months of age when hearing loss progressed more rapidly than males. Research showing these sex differences in hearing loss indicates similarities between rats and humans.

A rat strain that shows promise as a model for studying human hearing loss is the dark agouti (DA) rat [17]. One rationale for examining hearing loss in DA rats has to do with the long-known association between hearing loss and kidney disease; DA rats are known to be susceptible to kidney disease [18–24], indicating DA rats may also demonstrate susceptibility to hearing loss. For example, in humans there are over 20 known congenital disorders that involve both hearing loss and renal abnormalities, including Alport Syndrome, Branchio-oto-renal syndrome, and Fabry disease [25]. In a 2564-person study, Vilayur et al. found that more than half of patients with moderate chronic kidney disease had hearing loss of at least 25 decibels (dB) [26]. Additionally, a study by Gatland et al. found that patients with chronic renal failure have both high- and low-frequency hearing loss [27]. Therefore, with the potential risk for hearing loss, the DA rat may show usefulness for auditory function studies.

The aim of the present study was to assess responses to auditory stimuli in DA rats as a function of age to determine their potential usefulness in hearing studies. To our knowledge, there are no published data characterizing hearing in DA rats. We used auditory brainstem response (ABR) testing to measure hearing in male and female DA rats between 3 and 18 months of age. Because DA rats are more susceptible to stressor-induced kidney disease, we hypothesized that DA rats will exhibit hearing loss with age. Additionally, we hypothesized that the DA rats would display sex differences in hearing loss, as is observed in humans.

## Materials and methods

### Animals

Male and female DA rats were initially acquired from Taconic Biosciences, Inc. (Rensselaer, NY). Taconic Biosciences no longer maintains and sells the DA rat line, but they can be purchased from Envigo and Janvier labs. All rats used in the current report were obtained from the existing inbred DA rat colony at the Medical College of Wisconsin, and were derived from animals bred between 7 and 14 generations out from the original commercial source. All rats tested at 18 months of age were derived from breeding pairs less than 10 generations out from the original commercial source. Rats were provided with free access to chow (Purina, diet 5001) and drinking water, and were maintained on a 12-h light/dark cycle. A common medical issue in our DA colony is skin lesions due to unknown causes. Since the cause was unknown, rats diagnosed with skin lesions were not included in the study. All animal studies were conducted at the Medical College of Wisconsin. Animals were euthanized in the animal surgery facility in the Medical College of Wisconsin according to approved procedures by either compressed CO_2_ with thoracotomy or by isoflurane anesthesia with radical thoracotomy. The present study was carried out in accordance with the recommendations in *The National Research Council Guide for the Care and Use of Laboratory Animals*. All animal procedures were approved by the Institutional Animal Care and Use Committee of the Medical College of Wisconsin (Protocol Number: AUA00004621).

The study used a cross-sectional design, with different cohorts of rats tested for each age group. Although 29 of 128 rats were tested at more than one age interval, this was not a longitudinal study. All the female rats tested at 12 months died before they reached 18 months, so different 18-month-old female rats were tested.

### ABR setup

**Figure 1:**
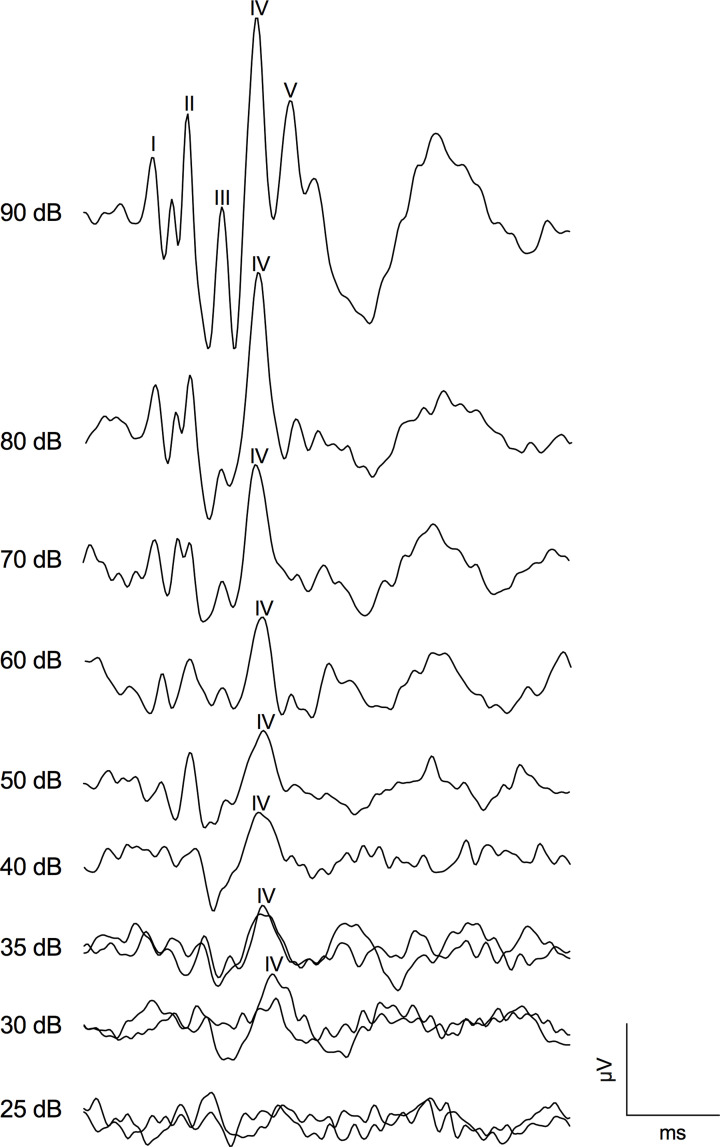
Example of ABR waveforms across stimulus level ABR recordings from a 3-month-old male DA rat with 32 kHz tone-burst stimuli are shown. Repeated recordings were conducted at 35-, 30- and 25 dB SPL to determine repeatability at the lower intensity levels. Hearing threshold was determined as 30 dB SPL, as this was the lowest intensity level showing a repeatable wave IV response. Abbreviation: SPL, sound pressure level.

ABR testing was used to evaluate hearing in DA rats as a function of age. This non-invasive method, which is extensively employed in both clinical and experimental studies, uses electrodes to detect electrical signals from the auditory brainstem pathway in response to acoustic signals. The resulting electrical recordings are displayed as ABR waveforms (Figure 1), with the waveform peaks corresponding to auditory structures along the peripheral auditory neural pathway (i.e., Wave I: auditory nerve, Wave II: cochlear nucleus, Wave III: superior olivary complex, Wave IV: lateral lemniscus, and Wave V: inferior colliculus) [28].

Rats were anesthetized by intraperitoneal injection of a mixture of ketamine hydrochloride (75 mg/kg) and xylazine hydrochloride (5–10 mg/kg) in sterile saline and were placed on a heated pad kept at 37°C in a sound-attenuated chamber during testing (Med Associates Inc, St. Albans, VT). Stainless steel 14 × 0.38 mm NeuroGuard needle electrodes (Consolidated Neuro Supply, Milford, OH) were placed subdermally in the base of the tail (ground), the vertex of the skull (noninverting) and behind the pinnae of the testing ear (inverting). Acoustic stimuli and simultaneous recordings were performed with a BioSig System III (Tucker-Davis Technologies (TDT), Alachua, FL). Anesthetized rats were exposed to acoustic stimuli consisting of a 5 ms, cosine-squared gated tone presented 21-times per second at the following typically studied frequencies in rats: 8, 16, and 32 kHz. The full frequency range of rat hearing is 0.2–80 kHz [29]. For each frequency, the tone level was presented beginning at the highest stimulation level of 90 dB sound pressure level (SPL), with the following stimulus levels presented in 5 dB decrements until reaching threshold or the 20 dB SPL stimulation level, which was the lowest stimulation level possible with the equipment. With each tone burst presentation, the phase was alternated 180 degrees to eliminate potential recording artifacts. Between 100 and 512 responses were averaged at each frequency and level combination. Acoustic stimuli were delivered to the test ear using a TDT EC1 speaker with plastic tubing connected to the speaker and placed directly in the ear canal. The TDT EC1 speaker was calibrated using a sweep from frequency range from 8 to 32 kHz, prior to testing. This was done with TDT SigCal software and a flat-response ACO Pacific microphone model 4016 (ACO Pacific, Inc, Belmont, CA) set up for close-field testing. The calibration generated a speaker response curve and a correction curve via FIR filter. Testing of animals occurred between 1 and 6 h after the rats entered their 12-h light period. Following ABR testing, atipamezole hydrochloride (0.5–1.0 mg/kg), an α2-adrenoreceptor antagonist and antidote of xylazine hydrochloride, was administered via intraperitoneal injection to reverse the anesthetic effects and shorten recovery time. Ketamine, xylazine, and atipamezole were obtained from Midwest Veterinary Supply (Lakeville, MN). All ABR testing procedures and measurements were performed by the same individual (author A.K.B.).

### ABR measurements

At three commonly tested audiometric frequencies in rats (8, 16, and 32 kHz), the hearing threshold was defined as the lowest intensity level where wave IV was identifiable and repeatable by visual inspection (Figure 1) [10], with higher threshold values indicating loss of hearing. ABR measurements were conducted on both the left and right ear for each animal and we report the threshold results for all tested ears. Latency of each waveform peak was measured for local maxima in milliseconds post-stimulus time. Interpeak latencies (IPLs) were calculated as the differences in latency between peaks. ABR waveform analyses were performed independently by the same two individuals (authors A.K.B. and C.L.R.), one of whom was blinded to rat age and sex (C.L.R.).

### Histology

Cochleae were processed similar to established methods, adjusting for specific requirements to access bone surrounding the cochleae [30]. Briefly, rat skulls were skinned and the auditory bullas were opened so the cochlea could be accessed. Neutral buffered formalin (10% v/v) (Thermo Fisher Scientific) was injected into the round window to allow fixation of the cochlea. The entire rat skulls were subsequently fixed in neutral buffered formalin (10% v/v). The samples were decalcified in Immunocal 1414-32 for 3–4 h until desired pliability. All samples were processed on a Sakura Tissue Tek VIP5 automated tissue processor to accomplish dehydration, clearing, and paraffin infiltration. At embedding, the half skulls were oriented with the interior of the skull placed down as to section from inside the cranial region and outward. The left ears of the rat samples were sectioned (conservatively) until the cochlea was reached. Additional surface decalcification with RDO solution (RDO Decalcifier, APEX Inc, EMS cat# 64143-01) was applied when needed. Embedded blocks were sectioned at 4 μm and placed on poly-l-lysine coated slides.

Hematoxylin and Eosin (H&E) staining was performed, with slides stained using the Sakura Prisma H&E Stainer. Images were carried out using a Hamamatsu NanoZoomer 2.0-HT digital slide scanner and analyzed with NDP.view software ver. 2.7.25 (Hamamatsu Photonics, Hamamatsu, Japan).

### Statistical analysis

All statistical analyses were performed with Prism 8 software (GraphPad, Inc.). A two-way ANOVA was used followed by multiple comparisons test, Tukey’s, Sidak’s, or Dunnett’s test, as indicated in the figure legend. Tukey’s test is a statistical analysis that compares the mean of every treatment with the mean of every other treatment; it was performed when comparing the thresholds within one sex between different ages. Sidak’s test corrects for type I errors; it was performed when comparing male with female at different ages. Dunnett’s test is used to compare multiple treatments with one control; it was performed when comparing differences between frequencies within one sex. Ear was treated as a variable for all statistical tests. The level of significance was *P*<0.05, where ns, not significant (*P*≥0.05), *P<0.05, **P<0.01, ***P<0.001, ****P<0.0001.

## Results and discussion

### Natural history of medical college of Wisconsin DA colony

**Figure 2:**
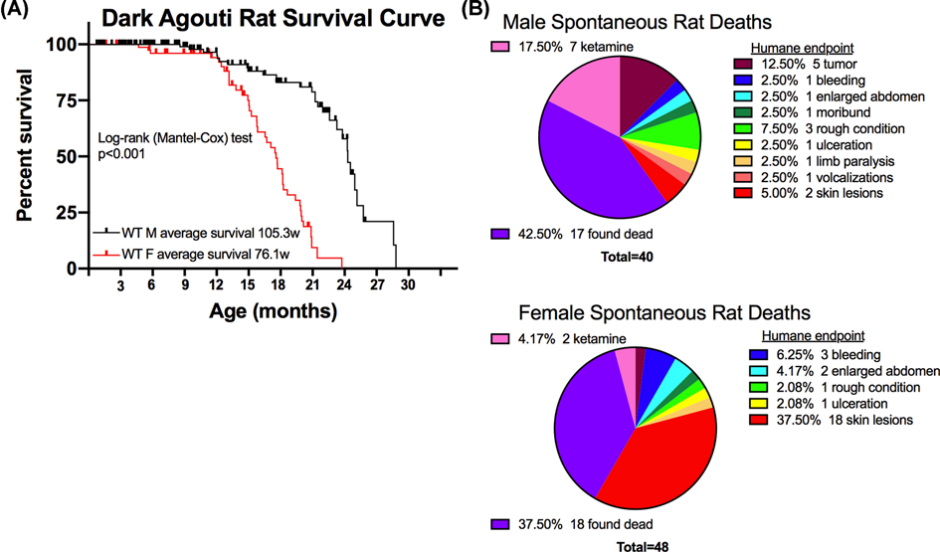
Survival curve and causes of spontaneous death of DA rat colony housed at the Medical College of Wisconsin (**A**) Contains the survival curve; rats were only counted as dead if they euthanized of natural causes or reached a humane endpoint that required them to be killed. (**B**) Displays spontaneous deaths, pie charts are shown to list the cause of death in male (top) and female (bottom) DA rats. Rats were either found dead of unknown reasons or reached a humane endpoint that required euthanasia. Two male rats died from ketamine-xylazine anesthesia use prior to the time when we initiated using the antidote atipamezole, and five male and two female rats died after exposure to ketamine-xylazine anesthesia followed by atipamezole treatment to shorten recovery time.

In our established DA rat colony, we noted a sex difference in the health of these animals over time, with female DA rats displaying more critical health conditions and an earlier mortality than male rats (Figure 2A). We identified spontaneous rat deaths to be due to a variety of causes (Figure 2B). One major cause of death for female, but not male, DA rats was from severe skin lesions that reached a point requiring humane euthanasia. The cause of the skin lesions has yet to be determined. These significant health issues contributed to the lack of females reaching the age of 18 months, therefore a lower N for this group.

One of the unexpected results from the present study was adverse reaction by DA rats to the ketamine-xylazine anesthesia. During ABR testing, animals need to be anesthetized to prevent movement and ensure the ABR recordings reflect only auditory responses. For our study, we carried out ABR measurements on both ears and at three frequencies which took an average of 45 min to complete. Therefore, sufficient anesthesia was needed to prevent animal movement for at least 45 min. Despite altering the dose of the ketamine-xylazine anesthesia, we observed that DA rats experienced prolonged recovery times and took an average of 3 h and 24 min to recover from anesthesia and be returned to their home cage. To aid with recovery, the antidote atipamezole was given which shortened the recovery time, allowing the animals to return to their home cage after an average of 1 h and 28 min.

A recent study by Giroux et al. revealed an age-dependent effect of ketamine-xylazine anesthesia in SD rats [31]. As SD rats age, they were observed to take longer to recover from the ketamine-xylazine anesthesia, and the older SD rats’ cardiac rate did not return to baseline level at the end of the 2-h test. Additionally, one 6-month and three 12-month SD rats were humanely euthanized after the test due to reaching humane endpoints [31]. Taken together, these results indicate careful monitoring is needed when administering ketamine-xylazine anesthesia, with the risk of occurrence of adverse events likely being strain-dependent.

### Hearing thresholds

**Figure 3:**
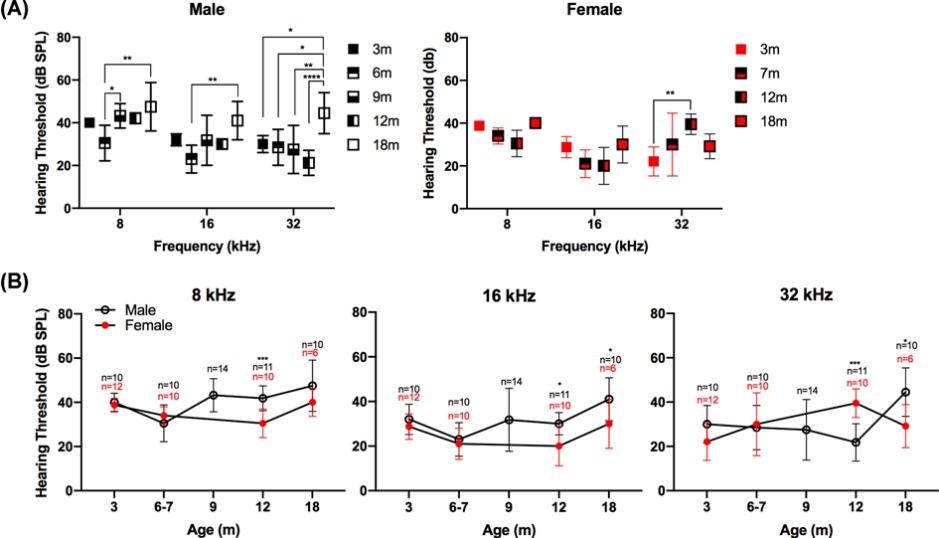
ABR testing in DA rats reveal differences in hearing thresholds between male and female rats with age (**A**) Displays the hearing thresholds by frequency across age for males (left) and females (right). (**B**) Displays the hearing thresholds across age comparing males (black) and females (red), for the three tested frequencies (1 SD). N indicates number of ears tested; to note, one male rat was tested at 12 months for the right ear only. This was a cross-sectional design study; the same rats were not always tested at each age. The female rats tested at 12 months died before reaching 18 months and were not included in the 18-month cohort. Multiple comparisons were run using Tukey’s test to compare ages for each sex, and Sidak’s test to compare male to female at each age. The level of significance was *P*<0.05, where *P<0.05, **P<0.01, ***P<0.001, ****P<0.0001.

The hearing thresholds of male and female DA rats at different ages were determined from the ABR waveforms. With increasing age, the male DA rat median hearing threshold increased from 3 and 18 months of age at 8, 16, and 32 kHz by 5, 10, and 10 dB respectively (Figure 3A, Supplementary Table S1). In contrast, age-dependent hearing loss was not observed in female DA rats. At the three tested frequencies, female DA rats did not exhibit consistent significant differences in hearing threshold between any of the ages other than from 3 to 12 months at 32 kHz (Figure 3A). Thus, within each sex, male rats exhibited stable hearing during the first year of life with hearing loss apparent by 18 months, whereas females exhibited stable hearing thresholds throughout their lifespan. However, the female lifespan is shorter than male lifespan, as discussed above (see Figure 2).

Examining by sex, at 18 months of age, male rats had higher hearing thresholds at frequencies of 16 and 32 kHz than female rats (Figure 3B). At 12 months of age, male rats had higher hearing thresholds at 8 and 16 kHz than female rats; however, at 32 kHz the female rats had higher thresholds. Comparing our results with Charlton et al. [15] and Balogová et al. [16] revealed that the males of the three strains of rats (LE, F344, and our DA rats) develop more severe hearing loss than female rats. Proposed causes for the hearing loss in male rats include effects from sex hormones including testosterone and estradiol [16]. Testosterone has been shown to damage hearing, whereas estradiol has been shown to protect hearing [32]. The sex differences in hearing loss across rat strains are consistent with what is observed in humans. Human population studies examining age-related hearing loss (ARHL) and sex have found significantly greater high-frequency hearing loss in male compared with female adults, with the significant differences persisting even when controlling for history of noise exposure and cardiovascular risk factors [33,34].

The hearing loss observed in male DA rats by 18 months of age demonstrates the usefulness of the DA male rat as a model for studying presbycusis. With the mean survival of male DA rats at 2 years (Figure 2), it is possible that these data are consistent with aging being associated with the observed hearing loss [35]. However, we cannot rule out the possibility that other factors contribute to the observed hearing loss in male DA rats. In contrast, female DA rats may be models for studies that require normal hearing throughout their typical lifespan. For the DA rats, the observed sex-dependent hearing difference with age might be unexpected, as female DA rats have a significantly shorter lifespan (median survival 76 weeks) than the males (median survival 105 weeks), and therefore might be expected to show ARHL at an earlier age than the male DA rats (Figure 2). However, these results were consistent with sex differences in hearing loss observed in humans and other rat strains.

### Absolute latency

**Figure 4:**
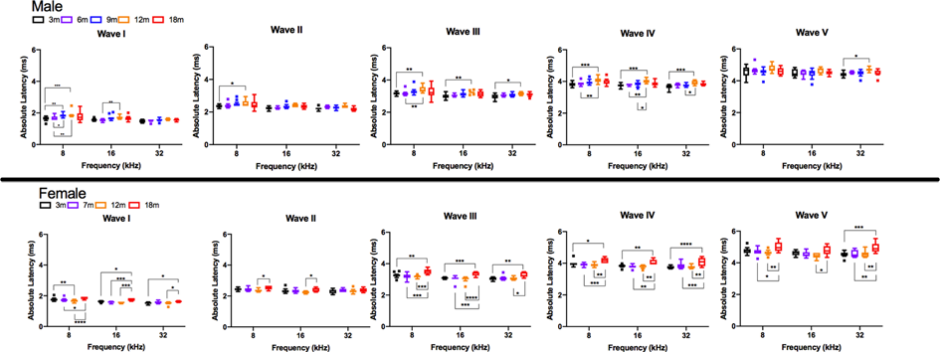
Analyses of absolute hearing latency revealed differences with age The absolute latencies at 90 dB SPL were determined for waves I through V for males and females for 8, 16, and 32 kHz across ages. Data are displayed in a box and whisker plot using Tukey’s method to show Tukey outliers. The line within the box denotes the median, the edges (hinges) of the box show the 25th and 75th percentiles, and the whiskers show the 25th percentile minus (1.5 interquartile range) and 75th percentile plus (1.5 interquartile range). Statistics analyzing effects of age within sex were conducted using two-way ANOVA using Tukey’s multiple comparison test where *P<0.05. The level of significance was *P*<0.05, where *P<0.05, **P<0.01, ***P<0.001, ****P<0.0001.

Absolute latencies were measured for waves I–V at 90 dB SPL for all ages and frequencies. For male rats, significant increases in absolute latency with age primarily occurred between young ages and 12 months, and most consistently between 3 and 12 months. From 3 to 12 months of age, significant increases in absolute latency were observed for wave I at 8 kHz; wave II at 8 kHz; wave III at 8, 16, and 32 kHz; wave IV at 8, 16, and 32 kHz; and wave V at 32 kHz (Figure 4, Supplementary Table S2). Female DA rats showed significant increases in absolute latencies primarily between young ages and 18 months. Significant increases in latency from 3 to 18 months of age were observed for wave I at 16 and 32 kHz; wave III at 8, 16, and 32 kHz; wave IV at 8, 16, and 32 kHz, and wave V at 32 kHz. Both male and female DA rats showed significantly longer wave III and IV latencies with age at all three tested frequencies, indicating potential for age-related neurological changes in the central auditory system with greater effects corresponding to the superior olivary complex and lateral lemniscus [36–38]. Longer absolute latencies with increasing age in the DA rats is consistent with previous reports in other rat models. In comparing the ABR responses of young (3–6 months) and aged (20–23 months) male F344 rats, Backoff and Caspary found significant increases in latency of waves I and V when compared at an equivalent dB SPL [12]. Further, they confirmed altered central auditory processing in the aged animals when tested at rapid stimulation rates [12].

**Figure 5:**
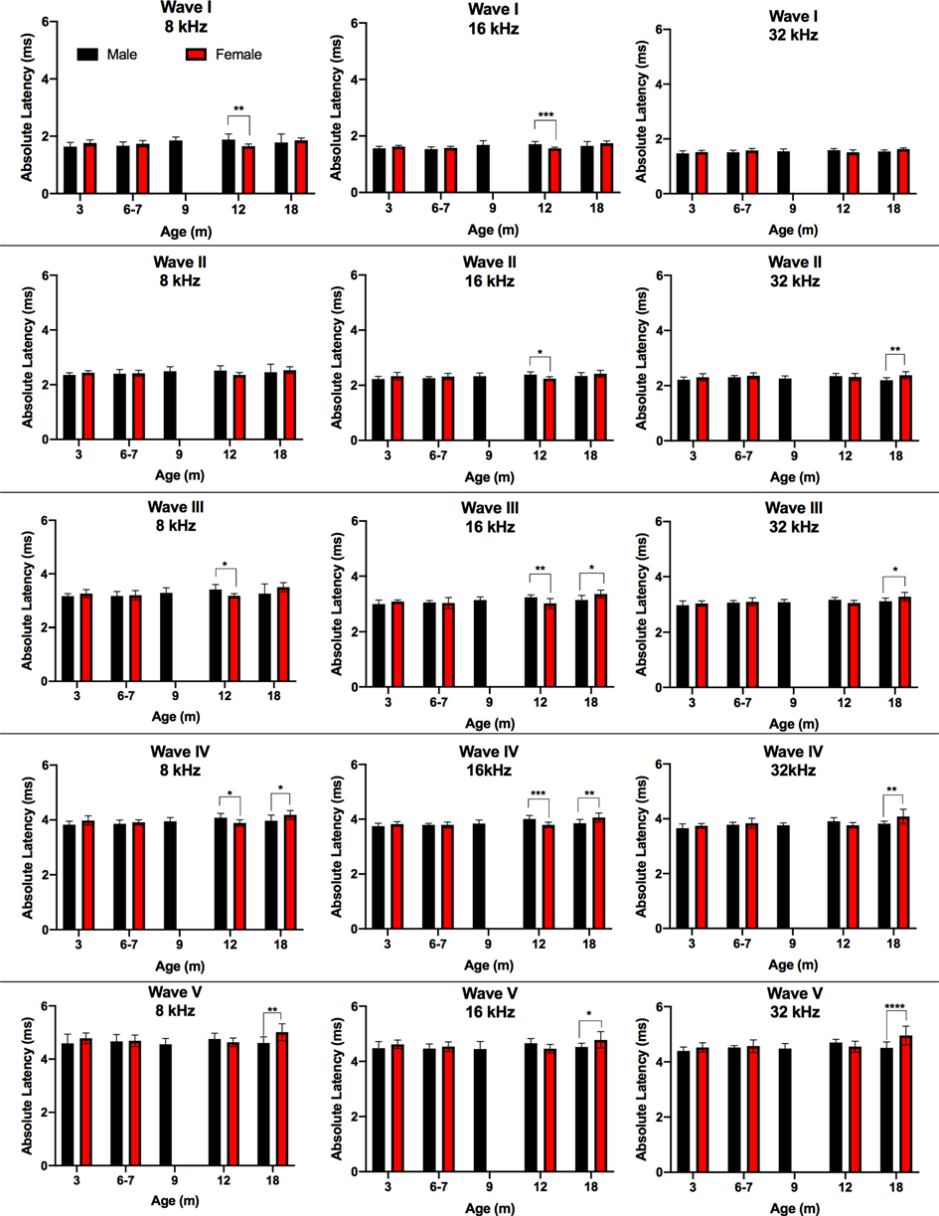
Analyses of absolute hearing latency revealed differences between sexes Bar graphs of absolute latency were plotted comparing male with female rats for 8, 16, and 32 kHz at the ages tested for waves I through V. Statistics comparing male with female rats at each age were conducted using two-way ANOVA using Sidak’s multiple comparison test where *P<0.05. The level of significance was *P*<0.05, where *P<0.05, **P<0.01, ***P<0.001, ****P<0.0001.

No sex differences in absolute latency were observed in young rats from 3 to 7 months, although sex differences appeared by 12 months of age (Figure 5). At the 12-month timepoint, male rats had significantly longer absolute latencies compared with female rats for wave I at 8 and 16 kHz, wave II at 16 kHz, wave III at 8 and 16 kHz, and wave IV at 8 and 16 kHz. In contrast, at 18 months of age, female rats had significantly longer absolute latencies compared with male rats for wave II for 32 kHz, wave III for 16 and 32 kHz, wave IV at 8, 16, and 32 kHz, and wave V at 8, 16, and 32 kHz. This is contrary to the findings by Church et al. comparing click-evoked ABRs between female and male SD rats, which showed significantly longer waveform peak latencies of waves II, III, and IV in the males compared with females [39]. They postulated that the shorter distances of the auditory pathway anatomical structures in females might account for the sex differences of absolute in the SD rat strain. While the exact reason for sex differences in absolute latency observed in DA rats was not clear, a similar pattern was observed in the statistically significant differences with age for 12-month males and 18-month females; therefore, the observed sex differences may be attributable to the within-sex age effects identified above.

### IPL

**Figure 6:**
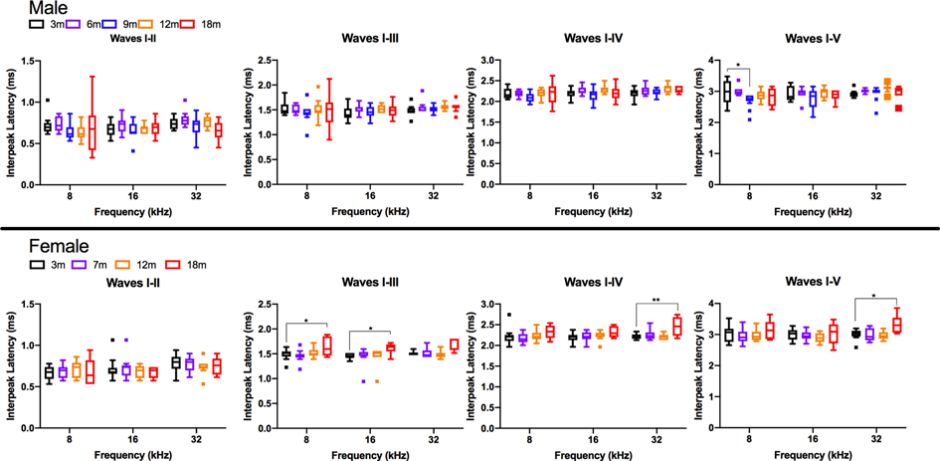
IPLs demonstrated that the latency from wave I to III, wave I to IV, and wave I to V increased with age in females, but not males IPL was calculated as the difference in latency from the wave I peak to the other designated peak for left and right ears at frequencies 8, 16, and 32 kHz. The average IPLs at 90 dB SPL are shown for specified ages of male DA rats (top panel) and female DA rats (bottom panel). Statistical analyses using two-way ANOVA and multiple comparisons with Dunnett’s test were performed. Data are displayed in a box and whisker plot using Tukey’s method to show Tukey outliers. The line within the box denotes the median, the edges (hinges) of the box show the 25th and 75th percentiles, and the whiskers show the 25th percentile minus (1.5 interquartile range) and 75th percentile plus (1.5 interquartile range). The level of significance was *P*<0.05, where *P<0.05, and **P<0.01.

In our DA rats, IPLs between ABR peak I and peaks II, III, IV, and V were calculated (Figure 6, Supplementary Table S3). Comparing age effects on IPLs within male and female DA rat groups revealed significant differences for male IPL I-V at 8 kHz between 3 and 6 months. Although there is a statistical significance, it is unclear if these results were meaningful given the lack of significance for all other measures and the large data spread for that 3-month interval (Figure 6). For females, IPLs were significantly longer between 3 and 18 months for I–III 8 kHz, I–III 16 kHz, I–IV 32 kHz, and I–V 32 kHz. Studies of age-related IPL changes in other rat strains include a comparison of male F344 and male LE rats [13]. Popelar et al. found significantly longer IPLs in the 1-month-old animals compared with12-month-old F344 and 24-month-old LE rats [13]. While they determined overall similar IPLs between rat strains, the F344 strain showed age-related increases in IPL at a younger age than the LE strain. Overbeck and Church compared IPLs of young adult SD and LE rats tested at ages ranging from 3 to 6 months [11]. Overall, they reported no significant differences in IPL between strains [11]. The significant increases in IPL for 18-month-old DA females were not accompanied by significant increases in threshold, indicating a suprathreshold increase in central auditory neural conduction time for the oldest female DA rats.

**Figure 7:**
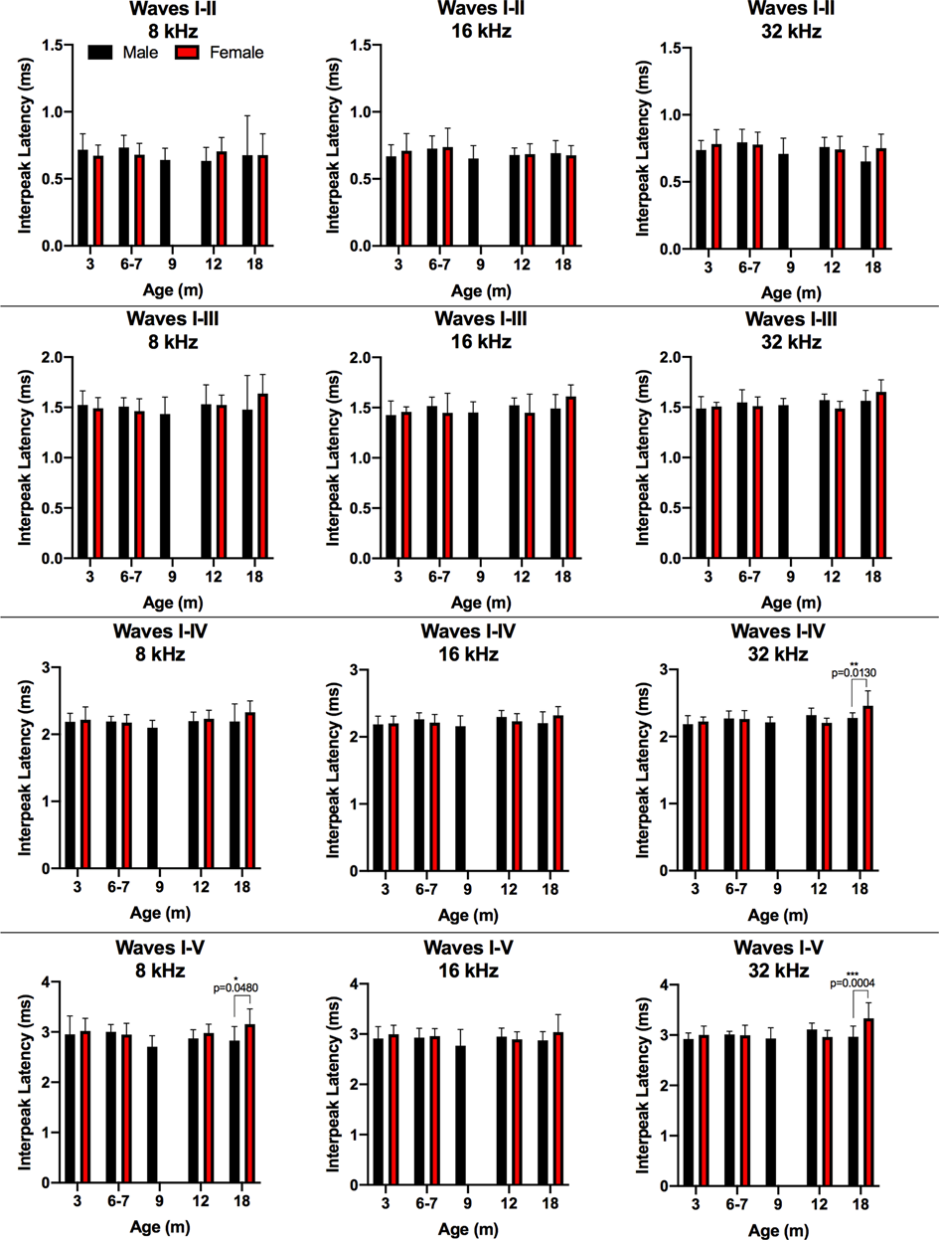
Analyses of IPLs revealed significantly longer IPLs for 18-month-old females at 8 kHz I–V and 32 kHz I–IV and I–V compared with males Male and female IPLs are shown for the ages tested (female rats were not tested at 9 months). Bar graphs show mean and 1 SD. Statistics comparing male with female rats at each age and frequency for the IPL were conducted by two-way ANOVA using Sidak’s multiple comparison test where *P<0.05. The level of significance was *P*<0.05, where *P<0.05 and ***P<0.001.

Comparing male with female IPLs across test frequency and age revealed significantly longer female IPLs at 18 months for 8 kHz IPL I–V, and for 32 kHz IPL I–IV and I–V compared with same-aged males (Figure 7, Supplementary Table S3). This may reflect the age-related increases in IPL observed only in the 18-month-old females, and not in the males. Similar to their findings of sex differences in absolute latency in SD rats, Church et al. found significantly longer IPLs for male SD rats which were also attributed to anatomical sex differences [11]. Gender differences in ABR interpeak intervals have been noted in humans with renal failure. A study by Antonelli et al. found that interpeak intervals I–III were prolonged in women with chronic renal failure, whereas men with chronic renal failure had less affected interpeak intervals I–III than women. The women with greatest effect on interpeak intervals I–III were women with better hearing [40].

### Cochlear histology

**Figure 8:**
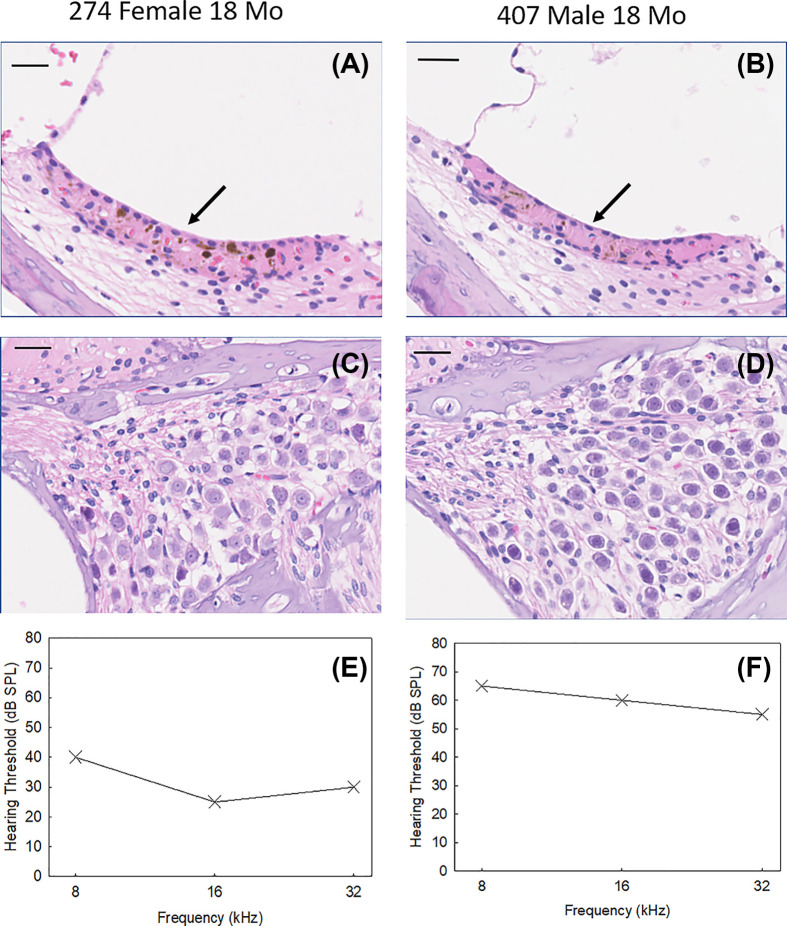
Histology of cochlea from aged DA rats reveal no obvious morphological defects Cross-sections of left cochlear SV (**A,B**) and SGCs (**C,D**) for 18-month-old DA rats female 274 (left panels) and male 407 (right panels). Left ear ABR hearing thresholds at 18 months for each animal are also shown (**E,F**). Arrows in (A,B) indicate marginal layer of SV. Scale bar = 25 microns.

Structural comparisons of the left ear middle cochlear turn were analyzed for 18-month-old female (274) and male (407) DA rats (Figure 8A–D). Despite the male having higher hearing thresholds ranging from 25 to 35 dB across frequencies compared with the female (Figure 8E,F); there were no morphological differences observed for stria vascularis (SV) or spiral ganglion cells (SGCs). It is possible that hearing threshold differences arose from differences in cochlear hair cell function, although hair cell counts and distortion product otoacoustic emission (DPOAE) measures were not performed for the present study, and therefore pose limitations on interpretation. Contrary to our findings in DA rat, previous studies in other rat strains have implicated the SV as a primary cause of hearing loss [13,16]. Balogova et al. found significant differences in DPOAE amplitude by sex in aged F344 rats, although no sex differences were found in number of surviving outer hair cells or number of ribbon synapses per inner hair cell. The main structural difference between sex in aged F344 rats were degenerative changes in SV marginal cells, with complete degenerative changes in 80% of males and full preservation in 70% of females [16].

In conclusion, the present study has demonstrated the usefulness of DA rats in hearing studies. For the first year of life, DA rats have similar hearing thresholds indicating the DA rats do not have ARHL for the first year. At 18 months, male DA rats have increased hearing thresholds, while female DA rats retain their hearing thresholds, but experience suprathreshold increases in absolute and IPLs.

## Supplementary Material

Supplementary Tables S1-S3

## Data Availability

Data requests can be emailed to any of the authors.

## Abbreviations

ABR: auditory brainstem response
ARHL: age-related hearing loss
DA: dark agouti
dB: decibel
DPOAE: distortion product otoacoustic emission
F344: Fischer 344 rat
H&E: Hematoxylin and Eosin
IPL: interpeak latency
LE: Long–Evans
SD: Sprague–Dawley
SPL: sound pressure level
SV: stria vascularis
TDT: Tucker-Davis Technologies
