# Supplementary material for: Auditory brainstem responses in aging dark agouti rats
Source: Biosci Rep. 2021 Feb 19;41(2):BSR20202724. doi: 10.1042/BSR20202724 (PMC7897922; doi:10.1042/BSR20202724)
Supplement: Supplementary Tables S1-S3 [file BSR-2020-2724_supp.pdf]

Supplemental Material

S1 Table. The mean and standard deviation of the hearing threshold of left and right ears at the three tested frequencies (8-, 16-, and 32 kHz) for DA rats at ages between 3-18 m.

Age	Sex	8kHz	N	16kHz	N	32kHz	N
3 m	Male	40.0 ± 4.1	10	32.0 ± 6.7	10	30.0 ± 8.5	10
3 m	Female	38.8 ± 3.1	12	28.8 ± 5.7	12	22.1 ± 8.4	12
6 m	Male	30.5 ± 8.3	10	23.0 ± 7.5	10	28.5 ± 10.0	10
(7 m)	Female	34.0 ± 3.9	10	21.0 ± 7.0	10	30.0 ± 14.1	10
9 m	Male	43.2 ± 7.5	14	31.8 ± 14.1	14	27.5 ± 13.7	14
12 m	Male	41.8 ± 5.6	11	30.0 ± 5.0	11	21.8 ± 8.4	11
12 m	Female	30.5 ± 6.4	10	20.0 ± 8.8	10	39.5 ± 6.4	10
18 m	Male	47.5 ± 11.6	10	41.0 ± 9.7	10	44.5 ± 10.9	10
18m	Female	40.0 ± 6.3	6	30.0 ± 11.0	6	29.2 ± 9.7	6

Wave	Frequency (kHz)	Age (month)	Male individual ears													
		3	1.80224	1.80224	1.72032	1.51552	1.72032	1.6384	1.31072	1.6384	1.59744	1.6384				
		6-7	1.80224	1.76128	1.55648	1.59744	1.96608	1.6384	1.59744	1.6384	1.55648	1.55648				
	8	9	1.67936	1.8432	1.8432	1.72032	1.80224	1.76128	1.88416	2.08896	1.92512	1.8432	1.8842	1.80224	2.08896	1.76128
		12	2.4576		1.80224	1.8432	1.80224	1.8432	1.8432	1.8432	1.80224	1.76128	1.8432	1.88416		
		18	1.843	2.41664	2.00704	1.51552	1.72032	1.8432	1.92512	1.55648	1.39264	1.59744				
		3	1.6384	1.72032	1.51552	1.51552	1.55648	1.55648	1.51552	1.55648	1.55648	1.47456				
		6-7	1.6384	1.59744	1.47456	1.6384	1.51552	1.47456	1.51552	1.59744	1.39	1.47456				
I	16	9	1.59744	1.72032	1.6384	1.51552	1.6384	1.6384	1.96608	2.048	1.55648	1.6384	1.7203	1.6384	1.6384	1.59744
		12	1.59744		1.6384	1.67936	1.80224	1.72032	1.59744	1.92512	1.72032	1.67936	1.6794	1.76128		
		18	1.67936	2.00704	1.59744	1.6384	1.67936	1.76128	1.4336	1.55648	1.55648	1.55648				
		3	1.59744	1.59744	1.4336	1.39264		1.47456	1.39264	1.47456	1.35168	1.55648				
		6-7	1.55648	1.47456	1.51552	1.55648	1.55648	1.55648	1.51552	1.55648	1.31072	1.51552				
	32	9	1.6384	1.59744	1.51552	1.59744	1.55648	1.51552		1.72032	1.35168	1.50766	1.5565	1.55648	1.51552	1.51552
		12	1.51552		1.55648	1.55648	1.59744	1.59744	1.51552	1.59744	1.67936	1.55648	1.6384	1.67936		
		18	1.55648	1.55648	1.55648	1.6384	1.55648	1.51552	1.4336	1.51552	1.59744	1.51552				
		3	2.41664	2.4576	2.33472	2.21184	2.49856	2.33472	2.33472	2.33472	2.33472	2.29376				
		6-7	2.41664	2.41664	2.29376	2.2528	2.78528	2.29376	2.29376	2.49856	2.41664	2.33472				
	8	9	2.37568	2.4576	2.37568	2.4576	2.37568	2.41664	2.49856	2.94912	2.49856	2.37568	2.5805	2.41664	2.74432	2.37568
		12	2.94912		2.37568	2.41664	2.49856	2.49856	2.37568	2.6624	2.37568	2.41664	2.4576	2.6624		
		18	2.417	3.072	2.4576	2.82624	2.048	2.53952	2.2528	2.37568	2.29376	2.29376				
		3	2.2528	2.33472	2.21184	2.048	2.37568	2.21184	2.21184	2.29376	2.12992	2.21184				
		6-7	2.21184	2.2528	2.17088	2.2528	2.2528	2.21184	2.29376	2.37568	2.29376	2.2528				
II	16	9	2.33472	2.33472	2.29376	2.33472	2.37568	2.2528	2.37568	2.6624	2.2528	2.2528	2.4576	2.29376	2.2528	2.21184
		12	2.29376		2.29376	2.29376	2.49856	2.37568	2.33472	2.53952	2.41664	2.29376	2.3757	2.53952		
		18	2.29376	2.53952	2.33472	2.49856	2.41664	2.41664	2.21184	2.2528	2.17088	2.2528				
		3	2.41664	2.2528	2.17088	2.048		2.17088	2.17088	2.21184	2.21184	2.2528				
		6-7	2.2528	2.21184	2.2528	2.33472	2.33472	2.2528	2.33472	2.41664	2.33472	2.33472				
	32	9	2.08896	2.41664	2.21184	2.33472	2.33472	2.2528		2.29376	2.2528	2.24111	2.3347	2.2528	2.2528	2.08896
		12	2.2528		2.29376	2.2528	2.4576	2.2528	2.29376	2.37568	2.33472	2.37568	2.4166	2.53952		
		18	2.21184	2.2528	2.37568	2.21184	2.21184	2.08896	2.21184	2.2528	2.048	2.08896				
		3	3.23584	3.19488	3.11296	2.99008	3.31776	3.11296	3.15392	3.19488	3.23584	3.072				
		6-7	3.19488	3.23584	3.03104	3.072	3.60448	3.072	3.11296	3.072	3.19488	3.15392				
	8	9	3.19488	3.2768	3.23584	3.23584	3.23584	3.19488	3.35872	3.8912	3.31776	3.23584	3.3997	3.23584	3.072	3.11296
		12	3.64544		3.19488	3.2768	3.39968	3.31776	3.31776	3.80928	3.2768	3.35872	3.3997	3.56352		
III		18	3.277	3.93216	3.31776	3.64	2.62144	3.4816	2.99008	3.15392	3.072	3.11296				
		3	2.99008	3.072	2.90816	2.744	3.2768	2.8672	2.94912	3.11296	2.94912	2.99008				
		6-7	3.19488	3.072	2.94912	2.99008	3.072	2.99008	3.072	3.03104	3.072	3.03104				
	16	9	3.15392	3.15392	3.11296	3.15392	3.19488	3.03104	3.19488	3.39968	3.03104	3.11296	3.2768	3.072	3.03104	2.94912
		12	3.19488		3.072	3.11296	3.39968	3.23584	3.23584	3.35872	3.23584	3.23584	3.2358	3.23584		

		18	3.11296	3.2768	3.15392	3.39968	3.15392	3.35872	2.99008	3.072	2.94912	2.90816				
	32	3	3.072	3.11296	2.8672	2.6624		2.90816	2.94912	3.19	2.8672	3.03104				
		6-7	3.072	2.94912	2.99008	3.11296	3.11296	3.03104	2.99008	3.03104	3.19488	3.11296				
		9	3.19488	3.11296	3.072	3.19488	3.11296	2.94912		3.2768	2.99008	2.97457	3.072	2.94912	3.03104	2.99008
		12	3.072		3.072	3.03104	3.2768	3.11296	3.15392	3.15392	3.23584	3.15392	3.2358	3.2768		
		18	3.15392	3.15392	3.15392	3.19488	3.11296	3.2768	2.99008	3.11296	3.072	2.8672				
IV	8	3	3.93216	3.85024	3.85024	3.60448	4.05504	3.85024	3.72736	3.80928	3.8912	3.6864				
		6-7	3.93216	3.93216	3.6864	3.85024	4.17792	3.6864	3.80928	3.80928	3.85024	3.85024				
		9	3.8912	3.76832	3.93216	4.01408	3.97312	3.93216	3.85024	4.3008	3.93216	3.93216	3.8502	3.97312	4.13696	3.80928
		12	4.42368		3.97312	3.80928	4.13696	3.97312	4.01408	4.17792	4.01408	4.05504	4.137	4.17792		
		18	4.055	4.42368	3.8912	4.13696	4.05504	3.93216	3.6864	3.85024	3.85024	3.85024				
	16	3	3.80928	3.6864	3.76832	3.52256	3.93216	3.72736	3.76832	3.76832	3.80928	3.6864				
		6-7	3.85024	3.80928	3.72736	3.76832	3.85024	3.6864	3.80928	3.76832	3.85024	3.80928				
		9	3.64544	3.8912	3.80928	3.93216	3.97312	3.80928	3.80928	4.05504	3.76832	3.80928	3.9322	3.80928	3.97312	3.56352
		12	3.93216		3.85024	3.85024	4.05504	3.93216	3.93216	4.17792	4.01408	3.97312	4.096	4.25984		
		18	3.85024	3.93216	3.80928	4.17792	3.72736	3.93216	3.76832	3.85024	3.64544	3.80928				
	32	3	3.80928	3.80928	3.56352	3.31776		3.60448	3.6864	3.72736	3.72736	3.6864				
		6-7	3.72736	3.60448	3.76832	3.8912	3.8912	3.6864	3.76832	3.80928	3.80928	3.85024				
		9	3.93216	3.76832	3.72736	3.8912	3.85024	3.6864		3.76832	3.6864	3.62653	3.7683	3.76832	3.72736	3.6864
		12	3.8912		3.72736	3.72736	4.096	3.8912	3.80928	3.80928	4.01408	3.93216	4.055	4.05504		
		18	3.72736	3.80928	3.80928	4.01408	3.76832	3.8912	3.72736	3.8912	3.80928	3.76832				
V	8	3	4.46464	4.79232	4.34176	3.8912	5.03808	4.3008	4.79	4.7104	4.95616	4.62848				
		6-7	4.75	4.62848	4.46464	4.54656	5.3248	4.62848	4.58752	4.7104	4.42368	4.62848				
		9	4.5056	3.932	4.58	4.58752	4.54656	4.62848	4.66944	4.874	4.66944	4.54656	4.7514	4.66944	4.464	4.38272
		12	5.20192		4.66944	4.62848	4.7104	4.54656	4.62848	4.99712	4.75136	4.75136	4.9562	4.464		
		18	4.588	4.91	4.54656	4.6	4.75136	4.95616	4.34	4.7104	4.38272	4.3008				
	16	3	4.42	4.46464		4.22	4.83328	4.628	4.178	4.38272	4.83328	4.34176				
		6-7	4.66944	4.62848	4.42368	4.096	4.66944	4.34176	4.5056	4.46464	4.38272	4.42368				
		9	4.46464	4.54656	4.58752	4.62848	4.79232	4.383	4.34176	4.25984	4.096	4.62848	4.4646	4.62848	4.71	3.768
		12	4.79232		4.464	4.383	4.87424	4.5056	4.75136	4.75136	4.7104	4.46	4.7104	4.833		
		18	4.54656	4.5056	4.58752	4.669	4.7104	4.54656	4.38272	4.58752	4.42368	4.25984				
	32	3	4.42368	4.5	4.25984	4.17792		4.38272	4.34176	4.66944	4.34176	4.46464				
		6-7	4.58752	4.46464	4.54656	4.62848	4.46464	4.46464	4.54656	4.54656	4.42368	4.54656				
		9	4.62848	4.34176	4.58752	4.7104	4.62848	4.46464		4.014	4.3008	4.48223	4.5875	4.5056	4.5056	4.5056
		12	4.66944		4.62848	4.54656	4.9152	4.7104	4.75136	4.7104	4.5056	4.7104	4.7514	4.833		
		18	4.014	4.63	4.42368	4.75136	4.42	4.66944	4.58752	4.62848	4.58752	4.38272				
Wave	Frequency (kHz)	Age (month)	Female individual ears													
I	8	3	1.72032	1.67936	1.80224	1.76128	1.76128	2.048	1.67936	1.6384	1.72032	1.80224	1.8432	1.72032		
		6-7	1.76128	1.80224	1.72032	1.67936	1.6384	1.67936	1.76128	1.67936	1.6384	2.00704				

		9														
		12	1.76128	1.59744	1.72032	1.67936	1.51552	1.6384	1.59744	1.6384	1.72032	1.67936				
		18	1.93	1.7203	1.8	1.93	1.88	1.88								
		3	1.59744	1.67936	1.6384	1.6384	1.6384	1.6384	1.55648	1.51552	1.6384	1.59744	1.6794	1.6384		
		6-7	1.59744	1.59744	1.55648	1.59744	1.47456	1.6384	1.6384		1.51552	1.59744				
	16	9														
		12	1.59744	1.59744	1.55648	1.59744	1.47456	1.51552	1.55648	1.55648	1.55648	1.59744				
		18	1.68	1.8432	1.64	1.72	1.72	1.84								
		3			1.51552	1.55648	1.51552	1.51552	1.39264	1.4336	1.51552	1.55648	1.5974	1.59744		
		6-7	1.55648	1.51552	1.51552	1.47456	1.51552	1.55648	1.6384	1.72032	1.6384	1.6384				
	32	9														
		12	1.55648	1.51552	1.55648	1.47456	1.51552	1.51552	1.51552	1.59744	1.26976	1.59744				
		18	1.6	1.67936	1.56	1.6	1.64	1.68								
		3	2.37568	2.41664	2.37568	2.41664	2.49856	2.58048	2.41664	2.41664	2.4576	2.49856	2.4986	2.29376		
		6-7	2.33472	2.41664	2.29376	2.29376	2.37568	2.41664	2.49856	2.49856	2.37568	2.6624				
	8	9														
		12	2.33472	2.4576	2.29376	2.29376	2.2528	2.37568	2.37568	2.37568	2.53952	2.29376				
		18	2.54	2.6624	2.33	2.46	2.54	2.66								
		3	2.2528	2.2528	2.2528	2.29376	2.70336	2.29376	2.2528	2.17088	2.4576	2.33472	2.3757	2.33472		
		6-7	2.2528	2.17088	2.29376	2.21184	2.53952	2.41664	2.37568		2.2528	2.33472				
II	16	9														
		12	2.2528	2.17088	2.29376	2.21184	2.17088	2.29376	2.29376	2.12992	2.33472	2.29376				
		18	2.42	2.49856	2.25	2.29	2.46	2.58								
		3			2.33472	2.49856	2.37568	2.08896	2.17088	2.17088	2.33472	2.33472	2.4576	2.2528		
		6-7	2.2528	2.41664	2.2528	2.2528	2.33472	2.4576	2.4576	2.53952	2.33472	2.2528				
	32	9														
		12	2.2528	2.41664	2.29376	2.2528	2.29376	2.29376	2.2528	2.12992	2.62144	2.33472				
		18	2.21	2.33472	2.29	2.38	2.46	2.58								
		3	3.23584	3.23584	3.19488	3.2768	3.2768	3.52256	3.11296	3.23584	3.23584	3.31776	3.48	2.94912		
		6-7	3.19488	3.2768	3.19488	3.072	2.82624	3.23584	3.23584	3.35872	3.11296	3.4816				
	8	9														
		12	3.19488	3.31776	3.19488	3.072	3.03104	3.19488	3.23584	3.19488	3.19488	3.15392				
		18	3.48	3.5635	3.28	3.36	3.52	3.77								
		3	3.03104	3.11296	3.072	2.99008	3.15392	3.11296	2.99008	3.03104	3.15392	3.11296	3.113	3.072		
III		6-7	3.072	2.53952	3.072	3.03104	3.03104	3.23584	3.15392		3.03104	3.072				
	16	9														
		12	3.072	2.53952	3.072	3.03104	2.90816	3.072	3.072	3.072	3.11296	3.15392				
		18	3.36	3.44064	3.28	3.11	3.44	3.48								
		3			2.99008	3.072	2.99008	2.99008	2.8672	2.90816	3.03104	3.15392	3.1539	3.11296		
	32	6-7	2.99008	2.94912	2.99008	2.94912	3.11296	3.11296	3.15392	3.44064	3.072	3.11296				
		9														

		12	2.99008	2.94912	3.03104	2.94912	3.03104	3.15392	3.03104	2.99008	3.23584	3.11296				
		18	3.19	3.2768	3.07	3.19	3.44	3.48								
		3	3.8912	3.8912	3.97312	3.76832	4.01408	4.01408	3.8912	3.93216	3.97312	4.01408	3.9322	4.46464		
		6-7	3.85024	3.93216	3.8912	3.72736	3.97312	3.93216	3.8912	4.05504	3.85024	4.01408				
	8	9														
		12	3.85024	4.096	3.8912	3.72736	3.72736	3.8912	3.93216	3.85024	4.01408	3.8912				
		18	4.18	4.1779	4.01	4.01	4.3	4.42								
		3	3.85024	3.80928	3.76832	3.60448	3.8912	3.85024	3.80928	3.85024	3.85024	3.97312	3.8502	3.76832		
		6-7	3.80928	3.56352	3.85024	3.76832	3.85024	3.97312	3.76832		3.76832	3.76832				
IV	16	9														
		12	3.80928	3.56352	3.85024	3.76832	3.6864	3.8912	3.8912	3.80928	3.85024	3.80928				
		18	3.97	4.01408	3.93	3.93	4.18	4.34								
		3			3.64544	3.6864	3.76832	3.6864	3.6864	3.6864	3.72736	3.8912	3.8502	3.80928		
		6-7	3.6864	3.64544	3.76832	3.6864	3.80928	3.93216	3.93216	4.25984	3.80928	3.85024				
	32	9														
		12	3.6864	3.64544	3.80928	3.6864	3.6864	3.85024	3.76832	3.72736	3.97312	3.80928				
		18	4.01	4.17792	3.73	3.85	4.3	4.42								
		3	4.83	4.75	4.79232	4.58752	4.95616	4.7104	4.75136	4.83328	4.87424	4.46	4.59	5.24288		
		6-7	4.58752	4.58752	4.62848	4.58752	4.25984	4.83328	4.79232	5.07904	4.7104	4.79232				
	8	9														
		12	4.58752	4.95616	4.62848	4.58752	4.34176	4.79232	4.66944	4.62848	4.5056	4.62848				
		18	5	4.9152	4.63	4.79	5.2	5.53								
		3	4.669	4.75	4.424	4.34	4.54	4.83328	4.7104	4.79232	4.62848	4.7	4.55	4.46464		
		6-7	4.464	4.3008	4.62	4.46464	4.42368	4.87424	4.7104		4.46464	4.5056				
V	16	9														
		12	4.464	4.3008	4.628	4.46464	4.13696	4.62848	4.5056	4.46464	4.34	4.62848				
		18	4.79	4.34176	4.71	5	5.2	4.63								
		3			4.46464	4.14	4.66944	4.42368	4.5056	4.5056	4.62848	4.54656	4.7923	4.54656		
		6-7	4.34176	4.34176	4.46464	4.38272	4.7104	4.79232	4.9152	4.87424	4.38272	4.5056				
	32	9														
		12	4.34176	4.34176	4.5056	4.38272	4.62848	4.7104	4.54656	4.5056	4.99712	4.54656				
		18	5.04	4.75	4.59	4.75	5.08	5.53								

Group	Waves	Frequency	Interpeak latencies												
3m male	I-II	8 kHz	0.6144	0.6144	0.77824	1.024	0.73728	0.65536	0.69632	0.69632	0.69632	0.65536			
		16 kHz	0.6144	0.69632	0.8192	0.69632	0.57344	0.6144	0.53248	0.65536	0.73728	0.73728			
		32 kHz	0.8192	0.73728		0.77824	0.86016	0.65536	0.65536	0.69632	0.73728	0.69632			
	I-III	8 kHz	1.4336	1.39264	1.59744	1.8432	1.6384	1.39264	1.47456	1.47456	1.55648	1.4336			
		16 kHz	1.35168	1.39264	1.72032	1.4336	1.39264	1.35168	1.22848	1.31072	1.55648	1.51552			
		32 kHz	1.47456	1.4336		1.55648	1.51552	1.51552	1.26976	1.4336	1.71544	1.47456			
	I-IV	8 kHz	2.12992	2.12992	2.33472	2.41664	2.29376	2.048	2.08896	2.21184	2.17088	2.048			
		16 kHz	2.17088	2.2528	2.37568	2.2528	2.2528	1.96608	2.00704	2.17088	2.21184	2.21184			

		32 kHz	2.21184	2.12992		2.29376	2.37568	2.21184	1.92512	2.12992	2.2528	2.12992			
	I-V	8 kHz	2.6624	2.62144	3.31776	3.47928	3.35872	2.99008	2.37568	2.6624	3.072	2.99008			
		16 kHz	2.7816		3.2768	2.66248	3.2768	2.74432	2.70448	3.07152	2.82624	2.8672			
		32 kHz	2.82624	2.82624		2.94912	2.99008	2.90256	2.78528	2.90816	3.19488	2.90816			
3m female	I-II	8 kHz	0.65536	0.57344	0.73728	0.73728	0.73728	0.65536	0.73728	0.65536	0.53248	0.77824	0.69632	0.57344	
		16 kHz	0.65536	0.6144	1.06496	0.69632	0.8192	0.69632	0.57344	0.65536	0.65536	0.65536	0.73728	0.69632	
		32 kHz		0.8192	0.86016	0.77824	0.8192	0.86016		0.94208	0.57344	0.73728	0.77824	0.65536	
	I-III	8 kHz	1.51552	1.39264	1.51552	1.4336	1.51552	1.6368	1.55648	1.51552	1.47456	1.59744	1.51552	1.2288	
		16 kHz	1.4336	1.4336	1.51552	1.4336	1.51552	1.4336	1.4336	1.35168	1.47456	1.51552	1.51552	1.4336	
		32 kHz		1.47456	1.47456	1.47456	1.51552	1.55648		1.51552	1.47456	1.47456	1.59744	1.51552	
	I-IV	8 kHz	2.17088	2.17088	2.2528	2.21184	2.2528	2.08896	2.21184	2.00704	1.96608	2.29376	2.21184	2.74432	
		16 kHz	2.2528	2.12992	2.2528	2.2528	2.21184	2.17088	2.12992	1.96608	2.21184	2.33472	2.37568	2.12992	
		32 kHz		2.12992	2.2528	2.29376	2.21184	2.2528		2.12992	2.17088	2.2528	2.33472	2.21184	
	I-V	8 kHz	3.10968	2.99008	3.19488	3.072	3.15392	2.7468	3.07064	2.82624	2.6624	3.19488	2.65776	3.52256	
		16 kHz	3.07156	2.7856	2.9016	3.15392	2.99008	2.87064	3.07064	2.7016	3.19488	3.2768	3.10256	2.82624	
		32 kHz		2.94912	3.15392	3.11296	3.11296	3.19488		2.58352	2.90816	3.072	2.99008	2.94912	
6m male	I-II	0.6144	0.73728	0.8192	0.69632	0.86016	0.65536	0.65536	0.65536	0.86016	0.77824				
		0.57344	0.69632	0.73728	0.77824	0.90376	0.65536	0.6144	0.73728	0.77824	0.77824				
		0.69632	0.73728	0.77824	0.8192	1.024	0.73728	0.77824	0.69632	0.86016	0.8192				
	I-III	1.39264	1.47456	1.6384	1.51552	1.6384	1.47456	1.47456	1.4336	1.4336	1.59744				
		1.55648	1.47456	1.55648	1.55648	1.682	1.47456	1.35168	1.51552	1.4336	1.55648				
		1.51552	1.47456	1.55648	1.47456	1.88416	1.47456	1.55648	1.47456	1.47456	1.59744				
	I-IV	2.12992	2.12992	2.21184	2.21184	2.29376	2.17088	2.2528	2.048	2.17088	2.29376				
		2.21184	2.2528	2.33472	2.29376	2.46024	2.21184	2.12992	2.21184	2.17088	2.33472				
		2.17088	2.2528	2.33472	2.2528	2.49856	2.12992	2.33472	2.12992	2.2528	2.33472				
	I-V	2.94776	2.90816	3.35872	2.99008	2.8672	2.8672	2.94912	2.99008	3.072	3.072				
		3.03104	2.94912	3.15392	2.99008	2.99272	3.03104	2.4576	2.8672	2.8672	2.94912				

		3.03104	3.03104	2.90816	3.03104	3.11296	2.99008	3.072	2.90816	2.99008	3.03104				
6m female	I-II	0.57344	0.57344	0.73728	0.73728	0.73728	0.6144	0.6144	0.73728	0.8192	0.65536				
		0.65536	0.73728	1.06496	0.73728	0.73728	0.57344	0.6144	0.77824		0.73728				
		0.69632	0.73728	0.8192	0.8192	0.69632	0.90112	0.77824	0.90112	0.8192	0.6144				
	I-III	1.4336	1.47456	1.18784	1.47456	1.47456	1.47456	1.39264	1.55648	1.67936	1.47456				
		1.47456	1.51552	1.55648	1.51552	1.51552	0.94208	1.4336	1.59744		1.47456				
		1.4336	1.47456	1.59744	1.51552	1.4336	1.4336	1.47456	1.55648	1.72032	1.47456				
	I-IV	2.08896	2.17088	2.33472	2.12992	2.21184	2.12992	2.048	2.2528	2.37568	2.00704				
		2.21184	2.29376	2.37568	2.12992	2.2528	1.96608	2.17088	2.33472		2.17088				
		2.12992	2.2528	2.29376	2.29376	2.17088	2.12992	2.21184	2.37568	2.53952	2.21184				
	I-V	2.82624	2.90816	2.62144	3.03104	3.072	2.78528	2.90816	3.15392	3.39968	2.78528				
		2.86656	3.06352	2.94912	3.072	2.94912	2.70336	2.8672	3.23584		2.90816				
		2.78528	2.94912	3.19488	3.2768	2.74432	2.82624	2.90816	3.23584	3.15392	2.8672				
9m male	I-II	0.69632	0.53248	0.57344	0.6144	0.57344	0.69632	0.65536	0.6144	0.73728	0.65536	0.86016	0.53248	0.6144	0.6144
		0.73728	0.65536	0.73728	0.4096	0.69632	0.73728	0.6144	0.6144	0.8192	0.6144	0.6144	0.6144	0.65536	0.6144
		0.45056	0.69632	0.77824		0.90112	0.77824	0.73728	0.8192	0.73728	0.73728	0.57344	0.73345574	0.69632	0.57344
	I-III	1.51552	1.39264	1.4336	1.47456	1.39264	1.51552	0.98304	1.4336	1.51552	1.4336	1.80224	1.39264	1.4336	1.35168
		1.55648	1.47456	1.55648	1.2288	1.47456	1.55648	1.39264	1.4336	1.6384	1.39264	1.35168	1.47456	1.4336	1.35168
		1.55648	1.55648	1.55648		1.6384	1.51552	1.51552	1.51552	1.59744	1.4336	1.55648	1.46691148	1.39264	1.47456
	I-IV	2.21184	2.08896	2.17088	1.96608	2.00704	1.96608	2.048	1.92512	2.29376	2.17088	2.21184	2.08896	2.17088	2.048
		2.048	2.17088	2.33472	1.8432	2.21184	2.21184	2.33472	2.17088	2.41664	2.17088	2.00704	2.17088	2.17088	1.96608
		2.29376	2.21184	2.29376		2.33472	2.21184	2.21184	2.17088	2.29376	2.17088	2.048	2.11887213	2.21184	2.17088
	I-V	2.82624	2.7368	2.74432	2.78528	2.74432	2.8672	2.37504	2.0888	2.8672	2.8672	2.78504	2.70336	2.8672	2.62144
		2.8672	2.94912	3.15392	2.37568	2.53952	2.74432	3.0716	2.82624	3.11296	2.7446	2.21184	2.99008	2.99008	2.17056
		2.99008	3.072	3.072		2.94912	3.03104	2.99008	2.74432	3.11296	2.94912	2.29368	2.97457049	2.94912	2.99008
12m male	I-II	0.49152	0.57344	0.69632	0.53248	0.57344	0.6144		0.57344	0.65536	0.8192	0.65536	0.77824		

		0.69632	0.65536	0.69632	0.73728	0.69632	0.69632		0.6144	0.65536	0.6144	0.6144	0.77824		
		0.73728	0.73728	0.86016	0.77824	0.65536	0.77824		0.69632	0.65536	0.77824	0.8192	0.86016		
	I-III	1.18784	1.39264	1.59744	1.47456	1.47456	1.55648		1.4336	1.47456	1.96608	1.59744	1.67936		
		1.59744	1.4336	1.59744	1.6384	1.51552	1.55648		1.4336	1.51552	1.4336	1.55648	1.47456		
		1.55648	1.51552	1.67936	1.6384	1.55648	1.59744		1.47456	1.51552	1.55648	1.59744	1.59744		
	I-IV	1.96608	2.17088	2.33472	2.17088	2.21184	2.29376		1.96608	2.12992	2.33472	2.29376	2.29376		
		2.33472	2.21184	2.2528	2.33472	2.29376	2.41664		2.17088	2.21184	2.2528	2.29376	2.49856		
		2.37568	2.17088	2.49856	2.29376	2.33472	2.41664		2.17088	2.29376	2.21184	2.37568	2.37568		
	I-V	2.74432	2.8672	2.90816	2.78528	2.94912	3.11296		2.78528	2.70336	3.15392	2.99008	2.57984		
		3.19488	2.8256	3.072	3.15392	2.99008	3.03104		2.70364	2.78528	2.82624	2.78064	3.07172		
		3.15392	3.072	3.31776	3.23584	2.82624	3.11296		2.99008	3.11296	3.11296	3.15392	3.15364		
12m female	I-II	0.57344	0.57344	0.73728	0.77824	0.8192	0.86016	0.6144	0.73728	0.73728	0.6144				
		0.65536	0.73728	0.69632	0.73728	0.77824	0.57344	0.6144	0.77824	0.57344	0.69632				
		0.69632	0.73728	0.77824	0.73728		0.90112	0.77824	0.77824	0.53248	0.73728				
	I-III	1.4336	1.47456	1.51552	1.6384	1.47456	1.72032	1.39264	1.55648	1.55648	1.47456				
		1.47456	1.51552	1.4336	1.51552	1.55648	0.94208	1.4336	1.55648	1.51552	1.55648				
		1.4336	1.47456	1.51552	1.51552		1.4336	1.47456	1.6384	1.39264	1.51552				
	I-IV	2.08896	2.17088	2.21184	2.33472	2.29376	2.49856	2.048	2.2528	2.21184	2.21184				
		2.21184	2.29376	2.21184	2.33472	2.29376	1.96608	2.17088	2.37568	2.2528	2.21184				
		2.12992	2.2528	2.17088	2.2528		2.12992	2.21184	2.33472	2.12992	2.21184				
	I-V	2.82624	2.90816	2.82624	3.072	2.78528	3.35872	2.90816	3.15392	2.99008	2.94912				
		2.86656	3.07152	2.6624	2.94912	2.78352	2.70336	2.8672	3.11296	2.90816	3.03104				
		2.78528	2.94912	3.11296	3.03104		2.82624	2.90816	3.19488	2.90816	2.94912				
18m male	I-II	0.5734377	0.45056	0.32768	0.32768	0.90112	0.65536	1.31072	0.69632	0.8192	0.69632				
		0.6144	0.73728	0.73728	0.77824	0.6144	0.53248	0.86016	0.65536	0.69632	0.69632				
		0.65536	0.8192	0.65536	0.77824	0.45056	0.69632	0.57344	0.57344	0.73728	0.57344				
	I-III	1.43359426	1.31072	0.90112	1.06496	1.67936	1.51552	2.12448	1.6384	1.59744	1.51552				

		1.4336	1.55648	1.47456	1.55648	1.39264	1.26976	1.76128	1.59744	1.51552	1.35168				
		1.59744	1.59744	1.55648	1.55648	1.47456	1.59744	1.55648	1.76128	1.59744	1.35168				
	I-IV	2.21183115	1.88416	2.33472	1.76128	2.4576	2.00704	2.62144	2.08896	2.29376	2.2528				
		2.17088	2.21184	2.048	2.33472	2.08896	1.92512	2.53952	2.17088	2.29376	2.2528				
		2.17088	2.2528	2.21184	2.29376	2.21184	2.2528	2.37568	2.37568	2.37568	2.2528				
	I-V	2.74430902	2.53952	3.03104	2.41488	2.99008	2.49336	3.08448	3.11296	3.15392	2.70336				
		2.8672	2.99008	3.03104	2.94912	2.8672	2.49856	3.0306	2.78528	3.03104	2.70336				
		2.45752	2.8672	2.86352	3.15392	2.99008	3.07352	3.11296	3.15392	3.11296	2.8672				
18m female	I-II	0.6144	0.53248	0.65536	0.94208	0.53248	0.77824								
		0.73728	0.6144	0.73728	0.65536	0.57344	0.73728								
		0.6144	0.73728	0.8192	0.65536	0.77824	0.90112								
	I-III	1.55648	1.47456	1.6384	1.8432	1.4336	1.88416								
		1.67936	1.6384	1.72032	1.59744	1.39264	1.6384								
		1.59744	1.51552	1.80224	1.59744	1.59744	1.80224								
	I-IV	2.2528	2.21184	2.41664	2.4576	2.08896	2.53952								
		2.29376	2.29376	2.4576	2.17088	2.21184	2.49856								
		2.41664	2.17088	2.6624	2.49856	2.2528	2.74432								
	I-V	3.072	2.82776	3.31776	3.19488	2.86488	3.64544								
		3.11296	3.072	3.4816	2.49856	3.2768	2.78528								
		3.44064	3.03104	3.44064	3.07064	3.15392	3.85024								

Comparison of Survival Curves			
Log-rank (Mantel-Cox) test			
Chi square		49.03	
df		1	
P value	<0.0001		
P value summary	****		
Are the survival curves sig different?	Yes		
Gehan-Breslow-Wilcoxon test			
Chi square		27.69	
df		1	
P value	<0.0001		
P value summary	****		
Are the survival curves sig different?	Yes		
Median survival			
M WT		105.3	
F WT		76.1	
Ratio (and its reciprocal)		-1	-1
95% CI of ratio	2.569e-322 to 6.084e-310		+infinity to +infinity
Hazard Ratio (Mantel-Haenszel)	A/B		B/A
Ratio (and its reciprocal)		0	+infinity
95% CI of ratio	2.569e-322 to 6.084e-310		+infinity to +infinity
Hazard Ratio (logrank)	A/B		B/A
Ratio (and its reciprocal)		0	+infinity
95% CI of ratio	2.569e-322 to 5.242e-310		+infinity to +infinity

Number of rows	333	333
# of blank lines	103	232
# rows with impossible data	0	0
# censored subjects	201	60
# deaths/events	29	41
Median survival	105.3	76.1

8 kHz					
Sidak's multiple comparisons test	Predicted (LS) mean diff.	95.00% CI of diff.	Significant ?	Summary	Adjusted P Value
Male - Female					
3	1.25	-6.001 to 8.501	No	ns	0.9868
6-7	-3.5	-11.07 to 4.073	No	ns	0.669
12	11.32	3.919 to 18.72	Yes	***	0.0008
18	7.5	-1.245 to 16.24	No	ns	0.1208
Tukey's multiple comparisons test	Predicted (LS) mean diff.	95.00% CI of diff.	Significant ?	Summary	Adjusted P Value
Male					
3 vs. 6-7	9.5	1.704 to 17.30	Yes	*	0.0106
3 vs. 12	-1.818	-9.435 to 5.799	No	ns	0.9227
3 vs. 18	-7.5	-15.30 to 0.2964	No	ns	0.0637
6-7 vs. 12	-11.32	-18.94 to -3.701	Yes	**	0.0012
6-7 vs. 18	-17	-24.80 to -9.204	Yes	****	<0.0001
12 vs. 18	-5.682	-13.30 to 1.935	No	ns	0.212
Female					
3 vs. 6-7	4.75	-2.714 to 12.21	No	ns	0.3448
3 vs. 12	8.25	0.7856 to 15.71	Yes	*	0.0245
3 vs. 18	-1.25	-9.967 to 7.467	No	ns	0.9816
6-7 vs. 12	3.5	-4.296 to 11.30	No	ns	0.6407
6-7 vs. 18	-6	-15.00 to 3.002	No	ns	0.3043
12 vs. 18	-9.5	-18.50 to -0.4975	Yes	*	0.0346
16 kHz					

Sidak's multiple comparisons test	Predicted (LS) mean diff.	95.00% CI of diff.	Significant?	Summary	Adjusted P Value
Male - Female					
3	3.25	-5.072 to 11.57	No	ns	0.7882
6-7	2	-6.692 to 10.69	No	ns	0.962
12	10	1.508 to 18.49	Yes	*	0.0144
18	11	0.9636 to 21.04	Yes	*	0.026
Tukey's multiple comparisons test	Predicted (LS) mean diff.	95.00% CI of diff.	Significant?	Summary	Adjusted P Value
Male					
3 vs. 6-7	9	0.05215 to 17.95	Yes	*	0.0481
3 vs. 12	2	-6.742 to 10.74	No	ns	0.9311
3 vs. 18	-9	-17.95 to -0.05215	Yes	*	0.0481
6-7 vs. 12	-7	-15.74 to 1.742	No	ns	0.1609
6-7 vs. 18	-18	-26.95 to -9.052	Yes	****	<0.0001
12 vs. 18	-11	-19.74 to -2.258	Yes	**	0.0078
Female					
3 vs. 6-7	7.75	-0.8169 to 16.32	No	ns	0.0903
3 vs. 12	8.75	0.1831 to 17.32	Yes	*	0.0435
3 vs. 18	-1.25	-11.25 to 8.754	No	ns	0.9876
6-7 vs. 12	1	-7.948 to 9.948	No	ns	0.9911
6-7 vs. 18	-9	-19.33 to 1.332	No	ns	0.1096
12 vs. 18	-10	-20.33 to 0.3321	No	ns	0.0614
32 kHz					
Sidak's multiple comparisons test	Predicted (LS) mean diff.	95.00% CI of diff.	Significant?	Summary	Adjusted P Value

Male - Female					
3	7.917	-2.757 to 18.59	No	ns	0.2262
6-7	-1.5	-12.65 to 9.648	No	ns	0.9948
12	-17.68	-28.57 to - 6.790	Yes	***	0.0004
18	15.33	2.461 to 28.21	Yes	*	0.013
Tukey's multiple comparisons test	Predicted (LS) mean diff.	95.00% CI of diff.	Significant ?	Summar y	Adjusted P Value
Male					
3 vs. 6-7	1.5	-9.976 to 12.98	No	ns	0.9859
3 vs. 12	8.182	-3.031 to 19.39	No	ns	0.2291
3 vs. 18	-14.5	-25.98 to - 3.024	Yes	**	0.0075
6-7 vs. 12	6.682	-4.531 to 17.89	No	ns	0.4034
6-7 vs. 18	-16	-27.48 to - 4.524	Yes	**	0.0026
12 vs. 18	-22.68	-33.89 to - 11.47	Yes	****	<0.0001
Female					
3 vs. 6-7	-7.917	-18.90 to 3.071	No	ns	0.2392
3 vs. 12	-17.42	-28.40 to - 6.429	Yes	***	0.0005
3 vs. 18	-7.083	-19.91 to 5.748	No	ns	0.4715
6-7 vs. 12	-9.5	-20.98 to 1.976	No	ns	0.1394
6-7 vs. 18	0.8333	-12.42 to 14.09	No	ns	0.9984
12 vs. 18	10.33	-2.918 to 23.59	No	ns	0.1792
Males					
Tukey's multiple comparisons test	Predicted (LS) mean diff.	95.00% CI of diff.	Significant ?	Summar y	Adjusted P Value
3m					
8 vs. 16	8	-3.595 to 19.60	No	ns	0.2309

8 vs. 32	10	-1.595 to 21.60	No	ns	0.1046
16 vs. 32	2	-9.595 to 13.60	No	ns	0.9103
6m					
8 vs. 16	7.5	-4.095 to 19.10	No	ns	0.2746
8 vs. 32	2	-9.595 to 13.60	No	ns	0.9103
16 vs. 32	-5.5	-17.10 to 6.095	No	ns	0.4953
9m					
8 vs. 16	11.43	1.629 to 21.23	Yes	*	0.0183
8 vs. 32	15.71	5.914 to 25.51	Yes	***	0.0008
16 vs. 32	4.286	-5.514 to 14.09	No	ns	0.5497
12m					
8 vs. 16	12.08	1.498 to 22.67	Yes	*	0.0214
8 vs. 32	20.83	10.25 to 31.42	Yes	****	<0.0001
16 vs. 32	8.75	-1.835 to 19.34	No	ns	0.1248
18m					
8 vs. 16	6.5	-5.095 to 18.10	No	ns	0.3767
8 vs. 32	3	-8.595 to 14.60	No	ns	0.8098
16 vs. 32	-3.5	-15.10 to 8.095	No	ns	0.7508
Tukey's multiple comparisons test	Predicted (LS) mean diff.	95.00% CI of diff.	Significant ?	Summar y	Adjusted P Value
8					
3m vs. 6m	9.5	-4.060 to 23.06	No	ns	0.2952
3m vs. 9m	-3.214	-15.77 to 9.340	No	ns	0.9519
3m vs. 12m	-2.083	-15.07 to 10.90	No	ns	0.9914
3m vs. 18m	-7.5	-21.06 to 6.060	No	ns	0.5346
6m vs. 9m	-12.71	-25.27 to -0.1597	Yes	*	0.0457

6m vs. 12m	-11.58	-24.57 to 1.400	No	ns	0.1026
6m vs. 18m	-17	-30.56 to -3.440	Yes	**	0.0069
9m vs. 12m	1.131	-10.80 to 13.06	No	ns	0.9989
9m vs. 18m	-4.286	-16.84 to 8.269	No	ns	0.8735
12m vs. 18m	-5.417	-18.40 to 7.566	No	ns	0.769
16					
3m vs. 6m	9	-4.560 to 22.56	No	ns	0.349
3m vs. 9m	0.2143	-12.34 to 12.77	No	ns	>0.9999
3m vs. 12m	2	-10.98 to 14.98	No	ns	0.9926
3m vs. 18m	-9	-22.56 to 4.560	No	ns	0.349
6m vs. 9m	-8.786	-21.34 to 3.769	No	ns	0.2963
6m vs. 12m	-7	-19.98 to 5.983	No	ns	0.5594
6m vs. 18m	-18	-31.56 to -4.440	Yes	**	0.0036
9m vs. 12m	1.786	-10.14 to 13.71	No	ns	0.9934
9m vs. 18m	-9.214	-21.77 to 3.340	No	ns	0.2511
12m vs. 18m	-11	-23.98 to 1.983	No	ns	0.1351
32					
3m vs. 6m	1.5	-12.06 to 15.06	No	ns	0.9979
3m vs. 9m	2.5	-10.05 to 15.05	No	ns	0.9806
3m vs. 12m	8.75	-4.233 to 21.73	No	ns	0.3335
3m vs. 18m	-14.5	-28.06 to -0.9395	Yes	*	0.0302
6m vs. 9m	1	-11.55 to 13.55	No	ns	0.9994
6m vs. 12m	7.25	-5.733 to 20.23	No	ns	0.5251
6m vs. 18m	-16	-29.56 to -2.440	Yes	*	0.0127
9m vs. 12m	6.25	-5.679 to 18.18	No	ns	0.5867
9m vs. 18m	-17	-29.55 to -4.445	Yes	**	0.0028
12m vs. 18m	-23.25	-36.23 to -	Yes	****	<0.0001

		10.27			
Females					
Tukey's multiple comparisons test	Predicted (LS) mean diff.	95.00% CI of diff.	Significant ?	Summar y	Adjusted P Value
3m					
8 vs. 16	10	0.1263 to 19.87	Yes	*	0.0466
8 vs. 32	16.67	6.793 to 26.54	Yes	***	0.0005
16 vs. 32	6.667	-3.207 to 16.54	No	ns	0.2411
7m					
8 vs. 16	13	2.184 to 23.82	Yes	*	0.0151
8 vs. 32	4	-6.816 to 14.82	No	ns	0.6454
16 vs. 32	-9	-19.82 to 1.816	No	ns	0.1198
12m					
8 vs. 16	10.5	-0.3161 to 21.32	No	ns	0.0587
8 vs. 32	-9	-19.82 to 1.816	No	ns	0.1198
16 vs. 32	-19.5	-30.32 to -8.684	Yes	***	0.0002
18m					
8 vs. 16	10	-3.964 to 23.96	No	ns	0.2033
8 vs. 32	10.83	-3.130 to 24.80	No	ns	0.1561
16 vs. 32	0.8333	-13.13 to 14.80	No	ns	0.9885
Tukey's multiple comparisons test	Predicted (LS) mean diff.	95.00% CI of diff.	Significant ?	Summar y	Adjusted P Value
8					
3m vs. 7m	4.75	-6.649 to 16.15	No	ns	0.6843
3m vs. 12m	8.25	-3.149 to 19.65	No	ns	0.2299
3m vs. 18m	-1.25	-14.56 to 12.06	No	ns	0.9944

7m vs. 12m	3.5	-8.405 to 15.41	No	ns	0.8612
7m vs. 18m	-6	-19.75 to 7.747	No	ns	0.6521
12m vs. 18m	-9.5	-23.25 to 4.247	No	ns	0.2669
16					
3m vs. 7m	7.75	-3.649 to 19.15	No	ns	0.2804
3m vs. 12m	8.75	-2.649 to 20.15	No	ns	0.1861
3m vs. 18m	-1.25	-14.56 to 12.06	No	ns	0.9944
7m vs. 12m	1	-10.91 to 12.91	No	ns	0.996
7m vs. 18m	-9	-22.75 to 4.747	No	ns	0.3123
12m vs. 18m	-10	-23.75 to 3.747	No	ns	0.226
32					
3m vs. 7m	-7.917	-19.32 to 3.482	No	ns	0.2628
3m vs. 12m	-17.42	-28.82 to -6.018	Yes	**	0.001
3m vs. 18m	-7.083	-20.39 to 6.227	No	ns	0.494
7m vs. 12m	-9.5	-21.41 to 2.405	No	ns	0.1597
7m vs. 18m	0.8333	-12.91 to 14.58	No	ns	0.9985
12m vs. 18m	10.33	-3.414 to 24.08	No	ns	0.2013

Male Wave I					
Tukey's multiple comparisons test	Predicted (LS) mean diff.	95.00% CI of diff.	Significant?	Summary	Adjusted P Value
8					
3m vs. 6m	-0.02867	-0.1964 to 0.1390	No	ns	0.9897
3m vs. 9m	-0.2136	-0.3688 to -0.05831	Yes	**	0.002
3m vs. 12m	-0.2458	-0.4096 to -0.08191	Yes	***	0.0005
3m vs. 18m	-0.1365	-0.3088 to 0.03577	No	ns	0.1898
6m vs. 9m	-0.1849	-0.3402 to -0.02964	Yes	*	0.0109
6m vs. 12m	-0.2171	-0.3809 to -0.05323	Yes	**	0.0032
6m vs. 18m	-0.1079	-0.2802 to 0.06444	No	ns	0.4194
9m vs. 12m	-0.03218	-0.1833 to 0.1189	No	ns	0.9766
9m vs. 18m	0.07705	-0.08317 to 0.2373	No	ns	0.674
12m vs. 18m	0.1092	-0.05933 to 0.2778	No	ns	0.3833
16					
3m vs. 6m	0.02894	-0.1388 to 0.1966	No	ns	0.9894
3m vs. 9m	-0.1217	-0.2770 to 0.03356	No	ns	0.1989
3m vs. 12m	-0.1486	-0.3124 to 0.01528	No	ns	0.0952
3m vs. 18m	-0.08238	-0.2547 to 0.08993	No	ns	0.6788
6m vs. 9m	-0.1506	-0.3059 to 0.004624	No	ns	0.0619
6m vs. 12m	-0.1775	-0.3414 to -0.01366	Yes	*	0.0265
6m vs. 18m	-0.1113	-0.2836 to 0.06099	No	ns	0.3865
9m vs. 12m	-0.02687	-0.1780 to 0.1242	No	ns	0.9881
9m vs. 18m	0.03933	-0.1209 to 0.1996	No	ns	0.9609
12m vs. 18m	0.0662	-0.1024 to 0.2348	No	ns	0.814
32					
3m vs. 6m	-0.03686	-0.2092 to	No	ns	0.9762

		0.1354			
3m vs. 9m	-0.07502	-0.2376 to 0.08760	No	ns	0.7074
3m vs. 12m	-0.1154	-0.2840 to 0.05312	No	ns	0.3264
3m vs. 18m	-0.06827	-0.2450 to 0.1085	No	ns	0.8232
6m vs. 9m	-0.03815	-0.1959 to 0.1196	No	ns	0.9629
6m vs. 12m	-0.07857	-0.2424 to 0.08528	No	ns	0.6764
6m vs. 18m	-0.0314	-0.2037 to 0.1409	No	ns	0.9869
9m vs. 12m	-0.04042	-0.1940 to 0.1132	No	ns	0.95
9m vs. 18m	0.006749	-0.1559 to 0.1694	No	ns	>0.9999
12m vs. 18m	0.04717	-0.1214 to 0.2157	No	ns	0.938
Male Wave II					
Tukey's multiple comparisons test	Predicted (LS) mean diff.	95.00% CI of diff.	Significant?	Summary	Adjusted P Value
8					
3m vs. 6m	-0.04506	-0.2104 to 0.1203	No	ns	0.9435
3m vs. 9m	-0.1375	-0.2906 to 0.01555	No	ns	0.1005
3m vs. 12m	-0.162	-0.3235 to -0.0004568	Yes	*	0.049
3m vs. 18m	-0.1024	-0.2678 to 0.06289	No	ns	0.4303
6m vs. 9m	-0.09245	-0.2455 to 0.06060	No	ns	0.4568
6m vs. 12m	-0.1169	-0.2784 to 0.04460	No	ns	0.2715
6m vs. 18m	-0.05738	-0.2227 to 0.1079	No	ns	0.8731
9m vs. 12m	-0.02447	-0.1734 to 0.1245	No	ns	0.9912
9m vs. 18m	0.03507	-0.1180 to 0.1881	No	ns	0.9695
12m vs. 18m	0.05954	-0.1020 to 0.2211	No	ns	0.8467
16					
3m vs. 6m	-0.02867	-0.1940 to 0.1367	No	ns	0.9892
3m vs. 9m	-0.1065	-0.2596 to 0.04656	No	ns	0.3105
3m vs. 12m	-0.1586	-0.3202 to	No	ns	0.0569

		0.002893			
3m vs. 18m	-0.1106	-0.2759 to 0.05473	No	ns	0.3506
6m vs. 9m	-0.07782	-0.2309 to 0.07523	No	ns	0.6259
6m vs. 12m	-0.13	-0.2915 to 0.03156	No	ns	0.1774
6m vs. 18m	-0.08192	-0.2472 to 0.08340	No	ns	0.6487
9m vs. 12m	-0.05213	-0.2011 to 0.09681	No	ns	0.8697
9m vs. 18m	-0.004096	-0.1572 to 0.1490	No	ns	>0.9999
12m vs. 18m	0.04804	-0.1135 to 0.2096	No	ns	0.9237
32					
3m vs. 6m	-0.09421	-0.2641 to 0.07564	No	ns	0.5436
3m vs. 9m	-0.04636	-0.2067 to 0.1139	No	ns	0.9306
3m vs. 12m	-0.1378	-0.3039 to 0.02838	No	ns	0.154
3m vs. 18m	0.01638	-0.1535 to 0.1862	No	ns	0.9989
6m vs. 9m	0.04785	-0.1076 to 0.2033	No	ns	0.9144
6m vs. 12m	-0.04356	-0.2051 to 0.1180	No	ns	0.9455
6m vs. 18m	0.1106	-0.05473 to 0.2759	No	ns	0.3506
9m vs. 12m	-0.09141	-0.2429 to 0.06003	No	ns	0.4576
9m vs. 18m	0.06274	-0.09275 to 0.2182	No	ns	0.7987
12m vs. 18m	0.1542	-0.007366 to 0.3157	No	ns	0.0691
Male Wave III					
Tukey's multiple comparisons test	Predicted (LS) mean diff.	95.00% CI of diff.	Significant?	Summary	Adjusted P Value
8					
3m vs. 6m	-0.01229	-0.2066 to 0.1820	No	ns	0.9998
3m vs. 9m	-0.1235	-0.3034 to 0.05645	No	ns	0.3245
3m vs. 12m	-0.2525	-0.4423 to -0.06260	Yes	**	0.0031
3m vs. 18m	-0.09778	-0.2921 to 0.09656	No	ns	0.6353
6m vs. 9m	-0.1112	-0.2911 to	No	ns	0.4331

		0.06874			
6m vs. 12m	-0.2402	-0.4300 to -0.05031	Yes	**	0.0056
6m vs. 18m	-0.08549	-0.2798 to 0.1088	No	ns	0.7427
9m vs. 12m	-0.129	-0.3041 to 0.04609	No	ns	0.2547
9m vs. 18m	0.02569	-0.1542 to 0.2056	No	ns	0.9948
12m vs. 18m	0.1547	-0.03518 to 0.3446	No	ns	0.1674
16					
3m vs. 6m	-0.06147	-0.2558 to 0.1329	No	ns	0.9062
3m vs. 9m	-0.1475	-0.3274 to 0.03243	No	ns	0.1626
3m vs. 12m	-0.2462	-0.4360 to -0.05629	Yes	**	0.0042
3m vs. 18m	-0.1516	-0.3459 to 0.04275	No	ns	0.2032
6m vs. 9m	-0.08602	-0.2659 to 0.09390	No	ns	0.679
6m vs. 12m	-0.1847	-0.3746 to 0.005179	No	ns	0.0608
6m vs. 18m	-0.09011	-0.2844 to 0.1042	No	ns	0.7035
9m vs. 12m	-0.09867	-0.2738 to 0.07641	No	ns	0.5277
9m vs. 18m	-0.004096	-0.1840 to 0.1758	No	ns	>0.9999
12m vs. 18m	0.09458	-0.09529 to 0.2844	No	ns	0.6442
32					
3m vs. 6m	-0.09748	-0.2971 to 0.1022	No	ns	0.6615
3m vs. 9m	-0.1086	-0.2970 to 0.07986	No	ns	0.5053
3m vs. 12m	-0.1991	-0.3944 to -0.003817	Yes	*	0.0433
3m vs. 18m	-0.1466	-0.3463 to 0.05303	No	ns	0.2577
6m vs. 9m	-0.01109	-0.1939 to 0.1717	No	ns	0.9998
6m vs. 12m	-0.1017	-0.2915 to 0.08822	No	ns	0.578
6m vs. 18m	-0.04915	-0.2435 to 0.1452	No	ns	0.9565
9m vs. 12m	-0.09056	-0.2686 to 0.08747	No	ns	0.6256
9m vs. 18m	-0.03806	-0.2208 to 0.1447	No	ns	0.9785

12m vs. 18m	0.0525	-0.1374 to 0.2424	No	ns	0.9406
Male Wave IV					
Tukey's multiple comparisons test	Predicted (LS) mean diff.	95.00% CI of diff.	Significant?	Summary	Adjusted P Value
8					
3m vs. 6m	-0.03277	-0.1934 to 0.1279	No	ns	0.9801
3m vs. 9m	-0.124	-0.2728 to 0.02468	No	ns	0.1496
3m vs. 12m	-0.2554	-0.4124 to -0.09850	Yes	***	0.0001
3m vs. 18m	-0.1475	-0.3081 to 0.01319	No	ns	0.0885
6m vs. 9m	-0.09128	-0.2400 to 0.05744	No	ns	0.4402
6m vs. 12m	-0.2227	-0.3796 to -0.06573	Yes	**	0.0013
6m vs. 18m	-0.1147	-0.2753 to 0.04595	No	ns	0.285
9m vs. 12m	-0.1314	-0.2761 to 0.01333	No	ns	0.0945
9m vs. 18m	-0.0234	-0.1721 to 0.1253	No	ns	0.9925
12m vs. 18m	0.108	-0.04895 to 0.2649	No	ns	0.3217
16					
3m vs. 6m	-0.04506	-0.2057 to 0.1156	No	ns	0.9376
3m vs. 9m	-0.09363	-0.2423 to 0.05510	No	ns	0.4137
3m vs. 12m	-0.2588	-0.4157 to -0.1018	Yes	***	0.0001
3m vs. 18m	-0.1024	-0.2630 to 0.05824	No	ns	0.4006
6m vs. 9m	-0.04857	-0.1973 to 0.1002	No	ns	0.8958
6m vs. 12m	-0.2137	-0.3707 to -0.05679	Yes	**	0.0022
6m vs. 18m	-0.05734	-0.2180 to 0.1033	No	ns	0.8613
9m vs. 12m	-0.1652	-0.3099 to -0.02044	Yes	*	0.0166
9m vs. 18m	-0.008774	-0.1575 to 0.1399	No	ns	0.9998
12m vs. 18m	0.1564	-0.0005526 to 0.3133	No	ns	0.0513
32					

3m vs. 6m	-0.1215	-0.2866 to 0.04353	No	ns	0.2553
3m vs. 9m	-0.1015	-0.2572 to 0.05429	No	ns	0.378
3m vs. 12m	-0.2507	-0.4122 to -0.08927	Yes	***	0.0003
3m vs. 18m	-0.1625	-0.3275 to 0.002566	No	ns	0.0559
6m vs. 9m	0.02005	-0.1310 to 0.1711	No	ns	0.9961
6m vs. 12m	-0.1292	-0.2862 to 0.02774	No	ns	0.1594
6m vs. 18m	-0.04096	-0.2016 to 0.1197	No	ns	0.9553
9m vs. 12m	-0.1493	-0.2964 to -0.002098	Yes	*	0.045
9m vs. 18m	-0.06101	-0.2121 to 0.09008	No	ns	0.7984
12m vs. 18m	0.08825	-0.06870 to 0.2452	No	ns	0.53
Male Wave V					
Tukey's multiple comparisons test	Predicted (LS) mean diff.	95.00% CI of diff.	Significant?	Summary	Adjusted P Value
8					
3m vs. 6m	-0.07792	-0.3371 to 0.1813	No	ns	0.9208
3m vs. 9m	0.03373	-0.2062 to 0.2737	No	ns	0.9951
3m vs. 12m	-0.1636	-0.4169 to 0.08960	No	ns	0.3864
3m vs. 18m	-0.01722	-0.2764 to 0.2420	No	ns	0.9997
6m vs. 9m	0.1116	-0.1283 to 0.3516	No	ns	0.7008
6m vs. 12m	-0.08573	-0.3390 to 0.1675	No	ns	0.8829
6m vs. 18m	0.0607	-0.1985 to 0.3199	No	ns	0.967
9m vs. 12m	-0.1974	-0.4309 to 0.03615	No	ns	0.14
9m vs. 18m	-0.05095	-0.2909 to 0.1890	No	ns	0.9769
12m vs. 18m	0.1464	-0.1068 to 0.3997	No	ns	0.5017
16					
3m vs. 6m	0.01742	-0.2489 to 0.2837	No	ns	0.9998
3m vs. 9m	0.02799	-0.2196 to 0.2756	No	ns	0.9979

3m vs. 12m	-0.1798	-0.4403 to 0.08068	No	ns	0.3185
3m vs. 18m	-0.04398	-0.3103 to 0.2223	No	ns	0.991
6m vs. 9m	0.01057	-0.2294 to 0.2505	No	ns	>0.9999
6m vs. 12m	-0.1972	-0.4505 to 0.05600	No	ns	0.2044
6m vs. 18m	-0.0614	-0.3206 to 0.1978	No	ns	0.9656
9m vs. 12m	-0.2078	-0.4413 to 0.02571	No	ns	0.1061
9m vs. 18m	-0.07196	-0.3119 to 0.1680	No	ns	0.9215
12m vs. 18m	0.1358	-0.1174 to 0.3891	No	ns	0.576
32					
3m vs. 6m	-0.1262	-0.3925 to 0.1401	No	ns	0.6859
3m vs. 9m	-0.08599	-0.3373 to 0.1653	No	ns	0.8788
3m vs. 12m	-0.3072	-0.5677 to -0.04667	Yes	*	0.012
3m vs. 18m	-0.1137	-0.3800 to 0.1526	No	ns	0.7631
6m vs. 9m	0.04024	-0.2035 to 0.2840	No	ns	0.991
6m vs. 12m	-0.1809	-0.4342 to 0.07229	No	ns	0.2841
6m vs. 18m	0.01251	-0.2467 to 0.2717	No	ns	>0.9999
9m vs. 12m	-0.2212	-0.4586 to 0.01625	No	ns	0.0808
9m vs. 18m	-0.02773	-0.2715 to 0.2161	No	ns	0.9979
12m vs. 18m	0.1935	-0.05978 to 0.4467	No	ns	0.2214
Female Wave I					
Tukey's multiple comparisons test	Predicted (LS) mean diff.	95.00% CI of diff.	Significant?	Summary	Adjusted P Value
8					
3m vs. 7m	0.02799	-0.05891 to 0.1149	No	ns	0.8344
3m vs. 12m	0.1099	0.02301 to 0.1968	Yes	**	0.0071
3m vs. 18m	-0.09202	-0.1935 to 0.009458	No	ns	0.0897
7m vs. 12m	0.08192	-0.008847 to 0.1727	No	ns	0.0921

7m vs. 18m	-0.12	-0.2248 to -0.01520	Yes	*	0.0181
12m vs. 18m	-0.2019	-0.3067 to -0.09712	Yes	****	<0.0001
16					
3m vs. 7m	0.0421	-0.04740 to 0.1316	No	ns	0.6098
3m vs. 12m	0.06076	-0.02614 to 0.1477	No	ns	0.2669
3m vs. 18m	-0.1192	-0.2207 to -0.01772	Yes	*	0.0145
7m vs. 12m	0.01866	-0.07460 to 0.1119	No	ns	0.9534
7m vs. 18m	-0.1613	-0.2683 to -0.05433	Yes	***	0.0009
12m vs. 18m	-0.18	-0.2848 to -0.07515	Yes	***	0.0001
32					
3m vs. 7m	-0.05735	-0.1481 to 0.03342	No	ns	0.3552
3m vs. 12m	0.008188	-0.08258 to 0.09896	No	ns	0.9954
3m vs. 18m	-0.1069	-0.2118 to -0.002139	Yes	*	0.0437
7m vs. 12m	0.06554	-0.02523 to 0.1563	No	ns	0.2403
7m vs. 18m	-0.0496	-0.1544 to 0.05521	No	ns	0.6052
12m vs. 18m	-0.1151	-0.2199 to -0.01033	Yes	*	0.0254
Female Wave II					
Tukey's multiple comparisons test	Predicted (LS) mean diff.	95.00% CI of diff.	Significant?	Summary	Adjusted P Value
8					
3m vs. 7m	0.02048	-0.1053 to 0.1463	No	ns	0.974
3m vs. 12m	0.07783	-0.04799 to 0.2036	No	ns	0.3742
3m vs. 18m	-0.09494	-0.2419 to 0.05198	No	ns	0.3351
7m vs. 12m	0.05734	-0.07407 to 0.1888	No	ns	0.6656
7m vs. 18m	-0.1154	-0.2672 to 0.03632	No	ns	0.1996
12m vs. 18m	-0.1728	-0.3245 to -0.02103	Yes	*	0.019

16					
3m vs. 7m	0.01479	-0.1148 to 0.1444	No	ns	0.9907
3m vs. 12m	0.0867	-0.03912 to 0.2125	No	ns	0.2792
3m vs. 18m	-0.08512	-0.2320 to 0.06181	No	ns	0.4331
7m vs. 12m	0.07191	-0.06311 to 0.2069	No	ns	0.5075
7m vs. 18m	-0.09991	-0.2548 to 0.05496	No	ns	0.3366
12m vs. 18m	-0.1718	-0.3236 to -0.02007	Yes	*	0.0199
32					
3m vs. 7m	-0.05325	-0.1847 to 0.07817	No	ns	0.7152
3m vs. 12m	-0.01229	-0.1437 to 0.1191	No	ns	0.9948
3m vs. 18m	-0.07383	-0.2256 to 0.07791	No	ns	0.5831
7m vs. 12m	0.04096	-0.09045 to 0.1724	No	ns	0.8475
7m vs. 18m	-0.02059	-0.1723 to 0.1312	No	ns	0.9846
12m vs. 18m	-0.06155	-0.2133 to 0.09020	No	ns	0.7146
Female Wave III					
Tukey's multiple comparisons test	Predicted (LS) mean diff.	95.00% CI of diff.	Significant?	Summary	Adjusted P Value
8					
3m vs. 7m	0.05721	-0.09868 to 0.2131	No	ns	0.7729
3m vs. 12m	0.07769	-0.07820 to 0.2336	No	ns	0.5637
3m vs. 18m	-0.2394	-0.4214 to -0.05735	Yes	**	0.0047
7m vs. 12m	0.02048	-0.1423 to 0.1833	No	ns	0.9877
7m vs. 18m	-0.2966	-0.4846 to -0.1086	Yes	***	0.0004
12m vs. 18m	-0.3171	-0.5051 to -0.1291	Yes	***	0.0002
16					
3m vs. 7m	0.05234	-0.1082 to 0.2129	No	ns	0.8294
3m vs. 12m	0.06827	-0.08762 to 0.2242	No	ns	0.663

3m vs. 18m	-0.2729	-0.4550 to -0.09090	Yes	***	0.0009
7m vs. 12m	0.01593	-0.1514 to 0.1832	No	ns	0.9946
7m vs. 18m	-0.3253	-0.5172 to -0.1334	Yes	***	0.0001
12m vs. 18m	-0.3412	-0.5292 to -0.1532	Yes	****	<0.0001
32					
3m vs. 7m	-0.06144	-0.2243 to 0.1014	No	ns	0.7577
3m vs. 12m	-0.02048	-0.1833 to 0.1423	No	ns	0.9877
3m vs. 18m	-0.2475	-0.4355 to -0.05951	Yes	**	0.0047
7m vs. 12m	0.04096	-0.1219 to 0.2038	No	ns	0.9127
7m vs. 18m	-0.1861	-0.3741 to 0.001931	No	ns	0.0535
12m vs. 18m	-0.227	-0.4151 to -0.03903	Yes	*	0.0112
Female Wave IV					
Tukey's multiple comparisons test	Predicted (LS) mean diff.	95.00% CI of diff.	Significant?	Summary	Adjusted P Value
8					
3m vs. 7m	0.06827	-0.08391 to 0.2205	No	ns	0.6457
3m vs. 12m	0.09285	-0.05933 to 0.2450	No	ns	0.3866
3m vs. 18m	-0.203	-0.3807 to -0.02532	Yes	*	0.0184
7m vs. 12m	0.02458	-0.1344 to 0.1835	No	ns	0.9776
7m vs. 18m	-0.2713	-0.4548 to -0.08777	Yes	**	0.0011
12m vs. 18m	-0.2959	-0.4794 to -0.1123	Yes	***	0.0003
16					
3m vs. 7m	0.03185	-0.1249 to 0.1886	No	ns	0.9513
3m vs. 12m	0.03003	-0.1221 to 0.1822	No	ns	0.9551
3m vs. 18m	-0.2378	-0.4155 to -0.06004	Yes	**	0.0039
7m vs. 12m	-0.00182	-0.1651 to 0.1615	No	ns	>0.9999
7m vs. 18m	-0.2696	-0.4569 to -0.08228	Yes	**	0.0016

12m vs. 18m	-0.2678	-0.4513 to -0.08425	Yes	**	0.0013
32					
3m vs. 7m	-0.09421	-0.2532 to 0.06474	No	ns	0.4126
3m vs. 12m	-0.02048	-0.1794 to 0.1385	No	ns	0.9868
3m vs. 18m	-0.3376	-0.5211 to -0.1540	Yes	****	<0.0001
7m vs. 12m	0.07373	-0.08522 to 0.2327	No	ns	0.6207
7m vs. 18m	-0.2434	-0.4269 to -0.05983	Yes	**	0.0043
12m vs. 18m	-0.3171	-0.5006 to -0.1336	Yes	***	0.0001
Female Wave V					
Tukey's multiple comparisons test	Predicted (LS) mean diff.	95.00% CI of diff.	Significant?	Summary	Adjusted P Value
8					
3m vs. 7m	0.09569	-0.1410 to 0.3324	No	ns	0.7167
3m vs. 12m	0.1489	-0.08778 to 0.3857	No	ns	0.3589
3m vs. 18m	-0.2294	-0.5058 to 0.04708	No	ns	0.1394
7m vs. 12m	0.05325	-0.1940 to 0.3005	No	ns	0.9428
7m vs. 18m	-0.325	-0.6105 to -0.03955	Yes	*	0.019
12m vs. 18m	-0.3783	-0.6638 to -0.09280	Yes	**	0.0043
16					
3m vs. 7m	0.0804	-0.1634 to 0.3242	No	ns	0.8244
3m vs. 12m	0.1607	-0.07604 to 0.3974	No	ns	0.292
3m vs. 18m	-0.1618	-0.4382 to 0.1146	No	ns	0.424
7m vs. 12m	0.08028	-0.1737 to 0.3343	No	ns	0.8421
7m vs. 18m	-0.2422	-0.5336 to 0.04920	No	ns	0.1383
12m vs. 18m	-0.3225	-0.6080 to -0.03697	Yes	*	0.0203
32					
3m vs. 7m	-0.04885	-0.2961 to	No	ns	0.955

		0.1984			
3m vs. 12m	-0.02837	-0.2756 to 0.2189	No	ns	0.9906
3m vs. 18m	-0.4344	-0.7199 to -0.1489	Yes	***	0.0008
7m vs. 12m	0.02048	-0.2268 to 0.2677	No	ns	0.9964
7m vs. 18m	-0.3855	-0.6710 to -0.1000	Yes	**	0.0035
12m vs. 18m	-0.406	-0.6915 to -0.1205	Yes	**	0.0019

Wave I 8 kHz					
Sidak's multiple comparisons test	Predicted (LS) mean diff.	95.00% CI of diff.	Significant?	Summary	Adjusted P Value
Male - Female					
3	-0.1263	-0.3023 to 0.04967	No	ns	0.2546
6-7	-0.06963	-0.2534 to 0.1142	No	ns	0.8059
12	0.2294	0.04981 to 0.4089	Yes	**	0.0067
18	-0.07498	-0.2872 to 0.1372	No	ns	0.8421
Wave I 16 kHz					
Sidak's multiple comparisons test	Predicted (LS) mean diff.	95.00% CI of diff.	Significant?	Summary	Adjusted P Value
Male - Female					
3	-0.06076	-0.1546 to 0.03306	No	ns	0.3504
6-7	-0.0476	-0.1483 to 0.05308	No	ns	0.65
12	0.1486	0.05284 to 0.2443	Yes	***	0.0007
18	-0.09394	-0.2071 to 0.01921	No	ns	0.1411
Wave I 32 kHz					
Sidak's multiple comparisons test	Predicted (LS) mean diff.	95.00% CI of diff.	Significant?	Summary	Adjusted P Value
Male - Female					
3	-0.04505	-0.1308 to 0.04074	No	ns	0.5557
6-7	-0.06554	-0.1490 to 0.01797	No	ns	0.1808
12	0.07857	-0.003020 to 0.1602	No	ns	0.0635
18	-0.08237	-0.1788 to 0.01406	No	ns	0.1231
Wave II 8 kHz					
Sidak's multiple comparisons test	Predicted (LS) mean diff.	95.00% CI of diff.	Significant?	Summary	Adjusted P Value
Male - Female					

3	-0.08192	-0.2517 to 0.08783	No	ns	0.6327
6-7	-0.01638	-0.1937 to 0.1609	No	ns	0.9988
12	0.1579	-0.01534 to 0.3311	No	ns	0.0877
18	-0.07443	-0.2792 to 0.1303	No	ns	0.828
Wave II 16 kHz					
Sidak's multiple comparisons test	Predicted (LS) mean diff.	95.00% CI of diff.	Significant?	Summary	Adjusted P Value
Male - Female					
3	-0.1031	-0.2188 to 0.01264	No	ns	0.0993
6-7	-0.05962	-0.1838 to 0.06456	No	ns	0.6369
12	0.1422	0.02416 to 0.2603	Yes	*	0.0118
18	-0.07761	-0.2172 to 0.06195	No	ns	0.5011
Wave II 32 kHz					
Sidak's multiple comparisons test	Predicted (LS) mean diff.	95.00% CI of diff.	Significant?	Summary	Adjusted P Value
Male - Female					
3	-0.09011	-0.2157 to 0.03547	No	ns	0.2543
6-7	-0.04915	-0.1714 to 0.07308	No	ns	0.7696
12	0.03537	-0.08405 to 0.1548	No	ns	0.9093
18	-0.1803	-0.3215 to -0.03919	Yes	**	0.0068
Wave III 8 kHz					
Sidak's multiple comparisons test	Predicted (LS) mean diff.	95.00% CI of diff.	Significant?	Summary	Adjusted P Value
Male - Female					
3	-0.09407	-0.3030 to 0.1149	No	ns	0.6899
6-7	-0.02458	-0.2428 to 0.1937	No	ns	0.9974
12	0.2361	0.02284 to 0.4493	Yes	*	0.024
18	-0.2357	-0.4877 to 0.01633	No	ns	0.0758

Wave III 16 kHz					
Sidak's multiple comparisons test	Predicted (LS) mean diff.	95.00% CI of diff.	Significant?	Summary	Adjusted P Value
Male - Female					
3	-0.09288	-0.2413 to 0.05553	No	ns	0.3841
6-7	0.02094	-0.1383 to 0.1802	No	ns	0.9953
12	0.2216	0.07011 to 0.3730	Yes	**	0.0015
18	-0.2142	-0.3932 to -0.03525	Yes	*	0.0125
Wave III 32 kHz					
Sidak's multiple comparisons test	Predicted (LS) mean diff.	95.00% CI of diff.	Significant?	Summary	Adjusted P Value
Male - Female					
3	-0.06471	-0.2014 to 0.07197	No	ns	0.6484
6-7	-0.02867	-0.1617 to 0.1044	No	ns	0.9698
12	0.1139	-0.01604 to 0.2439	No	ns	0.108
18	-0.1656	-0.3192 to -0.01198	Yes	*	0.0295
Wave IV 8 kHz					
Sidak's multiple comparisons test	Predicted (LS) mean diff.	95.00% CI of diff.	Significant?	Summary	Adjusted P Value
Male - Female					
3	-0.1543	-0.3180 to 0.009402	No	ns	0.0723
6-7	-0.05325	-0.2242 to 0.1177	No	ns	0.8935
12	0.194	0.02697 to 0.3610	Yes	*	0.0162
18	-0.2099	-0.4073 to -0.01245	Yes	*	0.0327
Wave IV 16 kHz					
Sidak's multiple comparisons test	Predicted (LS) mean diff.	95.00% CI of diff.	Significant?	Summary	Adjusted P Value
Male - Female					

3	-0.07509	-0.1989 to 0.04868	No	ns	0.4149
6-7	0.00182	-0.1310 to 0.1346	No	ns	>0.9999
12	0.2137	0.08744 to 0.3400	Yes	***	0.0002
18	-0.2104	-0.3597 to -0.06117	Yes	**	0.0023
Wave IV 32 kHz					
Sidak's multiple comparisons test	Predicted (LS) mean diff.	95.00% CI of diff.	Significant?	Summary	Adjusted P Value
Male - Female					
3	-0.08465	-0.2468 to 0.07752	No	ns	0.5613
6-7	-0.05734	-0.2152 to 0.1005	No	ns	0.8279
12	0.1456	-0.008620 to 0.2998	No	ns	0.0716
18	-0.2598	-0.4420 to -0.07749	Yes	**	0.0021
Wave V 8 kHz					
Sidak's multiple comparisons test	Predicted (LS) mean diff.	95.00% CI of diff.	Significant?	Summary	Adjusted P Value
Male - Female					
3	-0.1901	-0.4557 to 0.07540	No	ns	0.2566
6-7	-0.01652	-0.2939 to 0.2608	No	ns	0.9998
12	0.1225	-0.1485 to 0.3934	No	ns	0.6869
18	-0.4023	-0.7225 to -0.08202	Yes	**	0.0079
Wave V 16 kHz					
Sidak's multiple comparisons test	Predicted (LS) mean diff.	95.00% CI of diff.	Significant?	Summary	Adjusted P Value
Male - Female					
3	-0.1389	-0.3478 to 0.07004	No	ns	0.325
6-7	-0.0759	-0.2936 to 0.1418	No	ns	0.848
12	0.2016	-0.005379 to 0.4086	No	ns	0.0592
18	-0.2567	-0.5013 to -0.01203	Yes	*	0.036

Wave V 32 kHz					
Sidak's multiple comparisons test	Predicted (LS) mean diff.	95.00% CI of diff.	Significant?	Summary	Adjusted P Value
Male - Female					
3	-0.1265	-0.3473 to 0.09423	No	ns	0.4709
6-7	-0.04915	-0.2640 to 0.1657	No	ns	0.9626
12	0.1523	-0.05766 to 0.3622	No	ns	0.2449
18	-0.4472	-0.6953 to -0.1991	Yes	****	<0.0001

Male waves I-II					
Dunnett's multiple comparisons test	Predicted (LS) mean diff.	95.00% CI of diff.	Significant?	Summary	Adjusted P Value
8					
3m vs. 6m	-0.01638	-0.1448 to 0.1120	No	ns	0.9933
3m vs. 9m	0.07607	-0.04281 to 0.1949	No	ns	0.3223
3m vs. 12m	0.08378	-0.04167 to 0.2092	No	ns	0.2864
3m vs. 18m	0.041	-0.08740 to 0.1694	No	ns	0.8415
16					
3m vs. 6m	-0.05761	-0.1860 to 0.07080	No	ns	0.6284
3m vs. 9m	0.01521	-0.1037 to 0.1341	No	ns	0.9932
3m vs. 12m	-0.01005	-0.1355 to 0.1154	No	ns	0.9988
3m vs. 18m	-0.02458	-0.1530 to 0.1038	No	ns	0.9697
32					
3m vs. 6m	-0.05734	-0.1890 to 0.07427	No	ns	0.6455
3m vs. 9m	0.02865	-0.09556 to 0.1529	No	ns	0.9404
3m vs. 12m	-0.02233	-0.1511 to 0.1064	No	ns	0.9779
3m vs. 18m	0.08602	-0.04560 to 0.2176	No	ns	0.3013
Male Waves I-III					
Dunnett's multiple comparisons test	Predicted (LS) mean diff.	95.00% CI of diff.	Significant?	Summary	Adjusted P Value
8					
3m vs. 6m	0.01638	-0.1428 to 0.1756	No	ns	0.9969
3m vs. 9m	0.09011	-0.05729 to 0.2375	No	ns	0.3616
3m vs. 12m	-0.006703	-0.1623 to 0.1488	No	ns	0.9999
3m vs. 18m	0.04556	-0.1137 to 0.2048	No	ns	0.8854

16					
3m vs. 6m	-0.09041	-0.2496 to 0.06880	No	ns	0.4267
3m vs. 9m	-0.02578	-0.1732 to 0.1216	No	ns	0.9781
3m vs. 12m	-0.09759	-0.2531 to 0.05796	No	ns	0.3391
3m vs. 18m	-0.06557	-0.2248 to 0.09364	No	ns	0.6931
32					
3m vs. 6m	-0.06062	-0.2238 to 0.1026	No	ns	0.7562
3m vs. 9m	-0.03356	-0.1876 to 0.1204	No	ns	0.9507
3m vs. 12m	-0.0837	-0.2433 to 0.07593	No	ns	0.4922
3m vs. 18m	-0.077	-0.2402 to 0.08619	No	ns	0.5819
Male Waves I-IV					
Dunnett's multiple comparisons test	Predicted (LS) mean diff.	95.00% CI of diff.	Significa nt?	Summ ary	Adjusted P Value
8					
3m vs. 6m	-0.004096	-0.1472 to 0.1390	No	ns	0.9999
3m vs. 9m	0.08953	-0.04298 to 0.2220	No	ns	0.277
3m vs. 12m	-0.009681	-0.1495 to 0.1302	No	ns	0.9991
3m vs. 18m	-0.004095	-0.1472 to 0.1390	No	ns	0.9999
16					
3m vs. 6m	-0.07399	-0.2171 to 0.06914	No	ns	0.5098
3m vs. 9m	0.02809	-0.1044 to 0.1606	No	ns	0.9568
3m vs. 12m	-0.1102	-0.2501 to 0.02962	No	ns	0.1636
3m vs. 18m	-0.01638	-0.1595 to 0.1267	No	ns	0.9955
32					
3m vs. 6m	-0.08465	-0.2314 to 0.06205	No	ns	0.4081
3m vs. 9m	-0.02646	-0.1649 to 0.1120	No	ns	0.9687
3m vs. 12m	-0.1353	-0.2788 to 0.008217	No	ns	0.0705

3m vs. 18m	-0.09284	-0.2395 to 0.05386	No	ns	0.3283
Male Waves I-V					
Dunnett's multiple comparisons test	Predicted (LS) mean diff.	95.00% CI of diff.	Significant?	Summary	Adjusted P Value
8					
3m vs. 6m	-0.04925	-0.2896 to 0.1911	No	ns	0.9615
3m vs. 9m	0.2473	0.02481 to 0.4698	Yes	*	0.0242
3m vs. 12m	0.08212	-0.1527 to 0.3169	No	ns	0.7962
3m vs. 18m	0.1262	-0.1141 to 0.3665	No	ns	0.496
16					
3m vs. 6m	-0.01652	-0.2627 to 0.2297	No	ns	0.9992
3m vs. 9m	0.1447	-0.08425 to 0.3736	No	ns	0.3286
3m vs. 12m	-0.03626	-0.2771 to 0.2046	No	ns	0.9866
3m vs. 18m	0.03703	-0.2092 to 0.2832	No	ns	0.9866
32					
3m vs. 6m	-0.08937	-0.3357 to 0.1570	No	ns	0.7703
3m vs. 9m	-0.01098	-0.2434 to 0.2215	No	ns	0.9998
3m vs. 12m	-0.1917	-0.4327 to 0.04922	No	ns	0.1564
3m vs. 18m	-0.04409	-0.2904 to 0.2022	No	ns	0.9751
Female Waves I-II					
Dunnett's multiple comparisons test	Predicted (LS) mean diff.	95.00% CI of diff.	Significant?	Summary	Adjusted P Value
8					
3m vs. 7m	-0.007509	-0.1156 to 0.1005	No	ns	0.9971
3m vs. 12m	-0.03209	-0.1401 to 0.07597	No	ns	0.8307
3m vs. 18m	-0.00412	-0.1303 to 0.1221	No	ns	0.9996

16					
3m vs. 7m	-0.02731	-0.1386 to 0.08403	No	ns	0.8954
3m vs. 12m	0.02594	-0.08217 to 0.1341	No	ns	0.9012
3m vs. 18m	0.03413	-0.09211 to 0.1604	No	ns	0.8661
32					
3m vs. 7m	0.004096	-0.1085 to 0.1167	No	ns	0.9995
3m vs. 12m	0.0405	-0.07519 to 0.1562	No	ns	0.7475
3m vs. 18m	0.03159	-0.09844 to 0.1616	No	ns	0.8944
Female Waves I-III					
Dunnett's multiple comparisons test	Predicted (LS) mean diff.	95.00% CI of diff.	Significa nt?	Summ ary	Adjusted P Value
8					
3m vs. 6m	0.02922	-0.09513 to 0.1536	No	ns	0.9058
3m vs. 12m	-0.03222	-0.1566 to 0.09213	No	ns	0.879
3m vs. 18m	-0.1468	-0.2920 to -0.001617	Yes	*	0.0468
16					
3m vs. 6m	0.01024	-0.1179 to 0.1384	No	ns	0.9956
3m vs. 12m	0.007509	-0.1169 to 0.1319	No	ns	0.9981
3m vs. 18m	-0.1536	-0.2989 to -0.008317	Yes	*	0.0353
32					
3m vs. 6m	-0.004096	-0.1337 to 0.1255	No	ns	0.9997
3m vs. 12m	0.01911	-0.1140 to 0.1522	No	ns	0.9748
3m vs. 18m	-0.1447	-0.2944 to 0.004905	No	ns	0.0605
Female Waves I-IV					
Dunnett's multiple comparisons test	Predicted (LS) mean diff.	95.00% CI of diff.	Significa nt?	Summ ary	Adjusted P Value
8					

3m vs. 6m	0.04028	-0.09731 to 0.1779	No	ns	0.8364
3m vs. 12m	-0.01707	-0.1546 to 0.1205	No	ns	0.9839
3m vs. 18m	-0.1126	-0.2733 to 0.04802	No	ns	0.2359
16					
3m vs. 6m	-0.01024	-0.1520 to 0.1315	No	ns	0.9968
3m vs. 12m	-0.03072	-0.1684 to 0.1069	No	ns	0.9182
3m vs. 18m	-0.1195	-0.2802 to 0.04128	No	ns	0.1955
32					
3m vs. 6m	-0.03686	-0.1802 to 0.1065	No	ns	0.8781
3m vs. 12m	0.02139	-0.1259 to 0.1687	No	ns	0.974
3m vs. 18m	-0.2335	-0.3990 to -0.06792	Yes	**	0.003
Female Waves I-V					
Dunnett's multiple comparisons test	Predicted (LS) mean diff.	95.00% CI of diff.	Significant?	Summary	Adjusted P Value
8					
3m vs. 6m	0.0677	-0.1533 to 0.2887	No	ns	0.8176
3m vs. 12m	0.03903	-0.1820 to 0.2600	No	ns	0.9564
3m vs. 18m	-0.1373	-0.3954 to 0.1207	No	ns	0.4563
16					
3m vs. 6m	0.0383	-0.1894 to 0.2660	No	ns	0.9622
3m vs. 12m	0.09993	-0.1212 to 0.3210	No	ns	0.5871
3m vs. 18m	-0.04236	-0.3005 to 0.2158	No	ns	0.9647
32					
3m vs. 6m	0.008496	-0.2218 to 0.2388	No	ns	0.9995
3m vs. 12m	0.0399	-0.1967 to 0.2765	No	ns	0.9604
3m vs. 18m	-0.3285	-0.5944 to -0.06257	Yes	*	0.011

Waves I-II 8 kHz					
Sidak's multiple comparisons test	Predicted (LS) mean diff.	95.00% CI of diff.	Significant?	Summary	Adjusted P Value
Male - Female					
3	0.04437	-0.1115 to 0.2003	No	ns	0.9207
6-7	0.05325	-0.1096 to 0.2161	No	ns	0.8756
12	-0.07149	-0.2306 to 0.08760	No	ns	0.6913
18	-0.0007507	-0.1888 to 0.1873	No	ns	>0.9999
Waves I-II 16 kHz					
Sidak's multiple comparisons test	Predicted (LS) mean diff.	95.00% CI of diff.	Significant?	Summary	Adjusted P Value
Male - Female					
3	-0.04233	-0.1496 to 0.06494	No	ns	0.7818
6-7	-0.01202	-0.1271 to 0.1031	No	ns	0.9981
12	-0.00633	-0.1158 to 0.1031	No	ns	0.9998
18	0.01638	-0.1130 to 0.1458	No	ns	0.9959
Waves I-II 32 kHz					
Sidak's multiple comparisons test	Predicted (LS) mean diff.	95.00% CI of diff.	Significant?	Summary	Adjusted P Value
Male - Female					
3	-0.03351	-0.1427 to 0.07572	No	ns	0.8982
6-7	0.01638	-0.09230 to 0.1251	No	ns	0.992
12	0.01778	-0.09145 to 0.1270	No	ns	0.9893
18	-0.09948	-0.2250 to 0.02601	No	ns	0.1733
Waves I-III 8 kHz					
Sidak's multiple comparisons test	Predicted (LS) mean diff.	95.00% CI of diff.	Significant?	Summary	Adjusted P Value
Male - Female					

3	0.03222	-0.1596 to 0.2241	No	ns	0.988
6-7	0.04506	-0.1553 to 0.2454	No	ns	0.965
12	0.006703	-0.1891 to 0.2025	No	ns	>0.9999
18	-0.1602	-0.3915 to 0.07121	No	ns	0.2872
Waves I-III 16 kHz					
Sidak's multiple comparisons test	Predicted (LS) mean diff.	95.00% CI of diff.	Significant?	Summary	Adjusted P Value
Male - Female					
3	-0.03212	-0.1744 to 0.1102	No	ns	0.9644
6-7	0.06853	-0.08415 to 0.2212	No	ns	0.6921
12	0.07298	-0.07221 to 0.2182	No	ns	0.5965
18	-0.1201	-0.2917 to 0.05145	No	ns	0.2766
Waves I-III 32 kHz					
Sidak's multiple comparisons test	Predicted (LS) mean diff.	95.00% CI of diff.	Significant?	Summary	Adjusted P Value
Male - Female					
3	-0.01966	-0.1301 to 0.09079	No	ns	0.985
6-7	0.03686	-0.07064 to 0.1444	No	ns	0.8553
12	0.08316	-0.02489 to 0.1912	No	ns	0.1956
18	-0.08738	-0.2115 to 0.03676	No	ns	0.2713
Waves I-IV 8 kHz					
Sidak's multiple comparisons test	Predicted (LS) mean diff.	95.00% CI of diff.	Significant?	Summary	Adjusted P Value
Male - Female					
3	-0.02799	-0.2034 to 0.1474	No	ns	0.9901
6-7	0.01638	-0.1668 to 0.1996	No	ns	0.9989
12	-0.03537	-0.2143 to 0.1436	No	ns	0.978
18	-0.1365	-0.3481 to 0.07499	No	ns	0.3538

Waves I-IV 16 kHz					
Sidak's multiple comparisons test	Predicted (LS) mean diff.	95.00% CI of diff.	Significant?	Summary	Adjusted P Value
Male - Female					
3	-0.01434	-0.1462 to 0.1175	No	ns	0.9977
6-7	0.04942	-0.09206 to 0.1909	No	ns	0.8473
12	0.06516	-0.06937 to 0.1997	No	ns	0.6294
18	-0.1174	-0.2764 to 0.04159	No	ns	0.2298
Waves I-IV 32 kHz					
Sidak's multiple comparisons test	Predicted (LS) mean diff.	95.00% CI of diff.	Significant?	Summary	Adjusted P Value
Male - Female					
3	-0.03959	-0.1741 to 0.09494	No	ns	0.9111
6-7	0.008192	-0.1228 to 0.1391	No	ns	0.9997
12	0.1171	-0.01452 to 0.2487	No	ns	0.0999
18	-0.1802	-0.3314 to -0.02902	Yes	*	0.013
Waves I-V 8 kHz					
Sidak's multiple comparisons test	Predicted (LS) mean diff.	95.00% CI of diff.	Significant?	Summary	Adjusted P Value
Male - Female					
3	-0.06384	-0.3336 to 0.2059	No	ns	0.958
6-7	0.05311	-0.2286 to 0.3348	No	ns	0.9815
12	-0.1069	-0.3822 to 0.1683	No	ns	0.7914
18	-0.3274	-0.6527 to -0.002047	Yes	*	0.048
Waves I-V 16 kHz					
Sidak's multiple comparisons test	Predicted (LS) mean diff.	95.00% CI of diff.	Significant?	Summary	Adjusted P Value
Male - Female					

3	-0.08313	-0.3052 to 0.1389	No	ns	0.8122
6-7	-0.0283	-0.2597 to 0.2031	No	ns	0.9964
12	0.05306	-0.1670 to 0.2731	No	ns	0.955
18	-0.1625	-0.4226 to 0.09754	No	ns	0.3853
Waves I-V 16 kHz					
Sidak's multiple comparisons test	Predicted (LS) mean diff.	95.00% CI of diff.	Significant?	Summary	Adjusted P Value
Male - Female					
3	-0.08148	-0.2844 to 0.1214	No	ns	0.7703
6-7	0.01638	-0.1811 to 0.2139	No	ns	0.9992
12	0.1502	-0.04833 to 0.3487	No	ns	0.2094
18	-0.3659	-0.5939 to -0.1378	Yes	***	0.0004
